# Biomimetic and temporal-controlled nanocarriers with ileum transporter targeting for achieving oral administration of chemotherapeutic drugs

**DOI:** 10.1186/s12951-022-01460-3

**Published:** 2022-06-15

**Authors:** Wei Liu, Ying Han, Xin Xin, Liqing Chen, Yanhong Liu, Chao Liu, Xintong Zhang, Mingji Jin, Jingzhe Jin, Zhonggao Gao, Wei Huang

**Affiliations:** 1 State Key Laboratory of Bioactive Substance and Function of Natural Medicines, Institute of Materia Medica, Chinese Academy of Medical Sciences and Peking Union Medical College, Beijing, 100050 People’s Republic of China; 2 Beijing Key Laboratory of Drug Delivery Technology and Novel Formulations, Department of Pharmaceutics, Institute of Materia Medica, Chinese Academy of Medical Sciences and Peking Union Medical College, Beijing, 100050 People’s Republic of China; 3 Department of Oncology, The First Hospital of Dandong City, Dandong, Liaoning 118000 People’s Republic of China

**Keywords:** Oral delivery, Chemotherapy, Bile acids, Epithelial permeability, Lung cancer, Antitumor efficacy

## Abstract

**Background:**

Oral chemotherapy is preferred for patients with cancer owing to its multiple advantages, including convenience, better patient compliance, and improved safety. Nevertheless, various physical barriers exist in this route that hamper the development of oral chemotherapeutic formulations, including destruction of drugs in the gastrointestinal tract (GIT), low permeability in enterocytes, and short residence time in the intestine. To overcome these limitations, it is necessary to design an efficient oral drug delivery system with high efficacy and improved safety.

**Results:**

Herein, we designed novel glycocholic acid (GCA)-functionalized double layer nanoparticles (GCA-NPs), which can act via an endogenous pathway and in a temporally controlled manner in the intestine, to enhance the oral bioavailability of hydrophobic chemotherapeutic drugs such as paclitaxel (PTX). GCA-NPs were composed of quercetin (Qu)-modified liposomes (QL) coated with GCA-chitosan oligosaccharide conjugate (GCOS). The GCA-NPs thus prepared showed prolonged intestinal retention time and good GIT stability due to the presence of chitosan oligosaccharide (COS) and enhanced active transportation via intestinal apical sodium-dependent bile acid transporter (ASBT) due to the presence of GCA. GCA-NPs also efficiently inhibited intestinal P-gp induced by Qu. PTX-loaded GCA-NPs (PTX@GCA-NPs) had a particle size of 84 nm and an entrapment efficiency of 98% with good stability. As a result, the oral bioavailability of PTX was increased 19-fold compared to that of oral Taxol^®^ at the same dose. Oral PTX@GCA-NPs displayed superior antitumor efficacy and better safety than Taxol^®^ when administered intravenously.

**Conclusions:**

Our novel drug delivery system showed remarkable efficacy in overcoming multiple limitations and is a promising carrier for oral delivery of multiple drugs, which addresses several challenges in oral delivery in the clinical context.

**Graphical Abstract:**

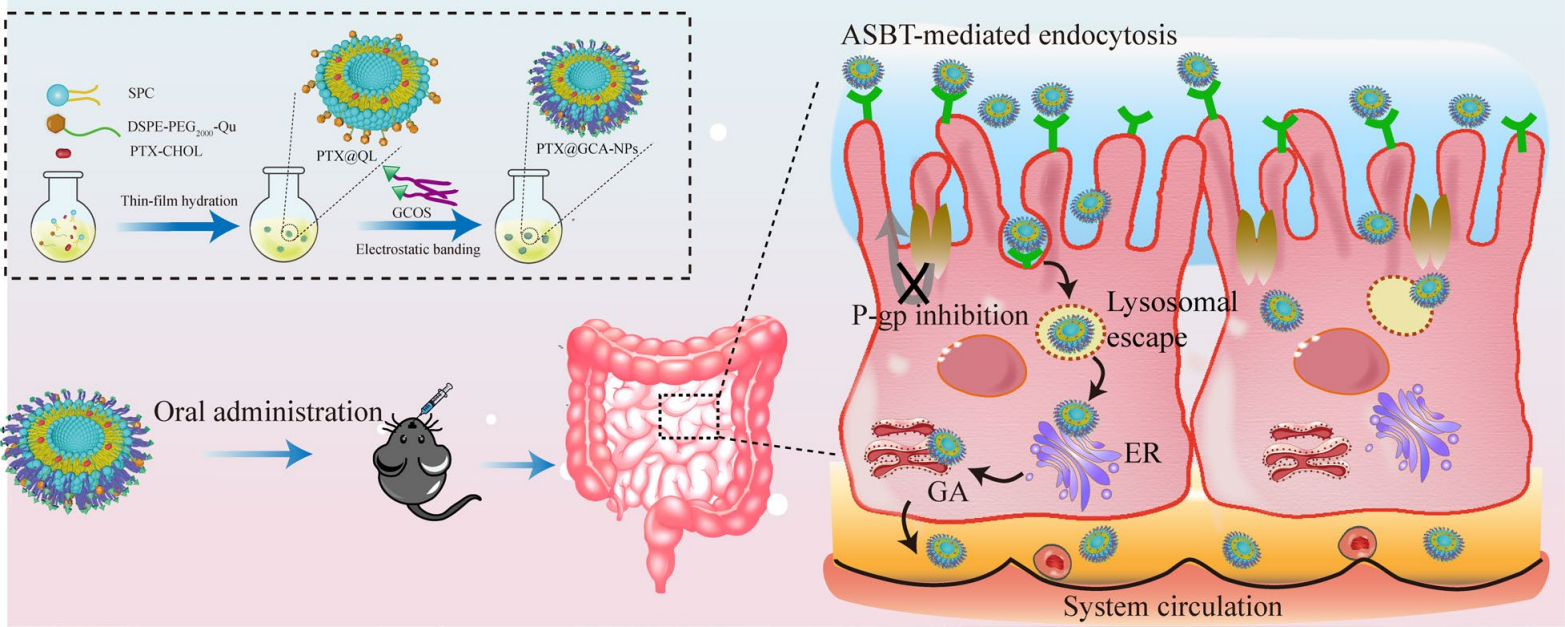

**Supplementary Information:**

The online version contains supplementary material available at 10.1186/s12951-022-01460-3.

## Introduction

Oral administration is one of the most convenient and acceptable routes for medication due to the reduced side effects as a result of the stable plasma concentration, ease of self-administration at home, and low risk of infection caused by long-term intravenous injections [1–4]. However, the clinical application of orally administered drugs is confronted with great challenges. For instance, a majority of drugs show undesirable physicochemical properties, such as poor water solubility and stability. Inadequate biopharmaceutical characteristics also limit the intestinal permeation and absorption of drugs [5]. In addition, there are complex biological barriers in the human body, including degradation of drugs in the gastrointestinal tract (GIT), transmembrane efflux transporters such as P-gp localized in enterocytes, low endocytosis efficiency, lysosomal destruction, and poor permeability of intestinal epithelial cells [6]. Therefore, simultaneously improving drug stability in the GIT and intestinal transport remains a key challenge. Nanoparticle-based drug delivery systems can overcome biological barriers in vivo after oral delivery [7, 8]. Numerous carriers can be applied to facilitate oral absorption, including enteric soluble materials and mucus penetration materials. Although various nanoparticle-based strategies have been studied, including micelles, nano-emulsions, solid lipid nanoparticles, nanofibers, and dendrimers [9–11], only a few oral nanoparticles have entered clinical research. This might be due to low GIT stability and poor therapeutic efficacy. Furthermore, it is difficult to achieve stable industrial production of many oral NPs [12].

Biomimetic strategies aim to improve the oral bioavailability of drugs by actively targeting intestinal transportation/receptors. Various transporters and receptors are expressed on the surface of enterocytes, such as neonatal Fc receptors (FcRn), folic acid receptors, transferrin receptors, oligopeptide transporters, sodium-dependent glucose receptors (SGLT), and apical sodium-dependent bile acid transporter (ASBT) [13, 14], which are considered potential targets for oral delivery [15]. In particular, ASBT is overexpressed in the distal ileum and pumps bile acids across apical membrane of the intestinal epithelial cells [13, 16–18]. Accordingly, ASBT mediates the enterohepatic circulation of bile acids, and approximately 95% of bile acids are reabsorbed and transported into the portal circulation system via the ASBT-mediated pathway [19]. Owing to its affinity for bile acids, ASBT plays a significant role in helping other moieties cross the intestinal epithelial barrier. As a natural substrate of ASBT, bile acids have attracted increasing attention for establishing bile acid-ASBT-mediated oral delivery formulations [19]. Numerous bile acid-decorated oral drug delivery strategies have been explored and have demonstrated the ability of ASBT to mediate enhanced oral drug absorption [13, 14, 17, 18, 20]. In our previous study, a cholic acid-decorated nanocomplex containing exendin-4 was shown to accumulate in the distal ileum, enhance plasma concentration, and consistently control blood glucose levels [21]. Therefore, we suggest ASBT as an ideal target for oral delivery. Bile acids are efficient substrates of ASBT and can be divided into free forms such as cholic acids (CA) and conjugate forms such as glycocholic acid (GCA). Conjugated bile acids possess better hydrophilicity and can be transported through ASBT with high efficiency [9, 12]. Therefore, we chose GCA as a ligand to target ASBT.

As mentioned above, we developed a GCA-ASBT-mediated active-targeting oral delivery system. Specifically, we conjugated GCA to oligosaccharide (COS) to obtain GCOS. COS is a low-molecular-weight chitosan with improved water solubility, intestinal temporal-controlled properties, and prolonged retention time in the GIT [12, 22]. Liposomes were chosen as the inner core of the drug carriers because of their good biocompatibility and well-established preparation process [23]. To inhibit P-gp efflux, liposomes were covalently decorated with the natural flavonoid, quercetin (Qu) [24–26]. Such a strategy could maintain drug encapsulation efficiency and achieve the co-delivery of a P-gp inhibitor. Finally, the surface of the liposomes was coated with GCOS using electrostatic interactions to obtain the desired oral nanoparticles (GCA-NPs). The GCA-NPs exhibited enhanced intestinal transportation through a combination of biomimetic ASBT-mediated pathway and intestinal P-gp inhibition, good stability in the GIT, and prolonged absorption time in the intestine.

To verify the oral efficacy of GCA-NPs, we chose lung cancer as a disease model and paclitaxel (PTX) as a model drug. Lung cancer has been reported as the leading cause of malignant tumor-related deaths worldwide in 2020, particularly non-small cell lung cancer (NSCLC), which is responsible for 85% of these cases [27, 28]. The 5-year survival rate of lung cancer after diagnosis was approximately 10–20% during 2010–2014 [27]. Intravenous injection of taxane-based chemotherapeutics remains the most frequently employed regimen for NSCLC treatment [29, 30]. However, intravenously administered taxane can cause severe side effects and is rapidly metabolized, greatly reducing the quality of life of patients [1, 31, 32]. Therefore, much effort has been devoted to the development of oral PTX formulations. Nevertheless, PTX, a hydrophobic molecule, is a typical substrate of intestinal P-gp, with poor oral bioavailability (less than 1%) [31]. Despite the application of numerous nanoparticles being reported for the delivery of oral PTX, the potential of such strategies has not been fully exploited [33–35]. For instance, the above oral PTX nanocarriers cannot overcome multiple gastrointestinal physiochemical barriers simultaneously, and it is difficult to achieve good oral bioavailability of PTX and concomitant antitumor efficiency in vivo.

**Fig. 1:**
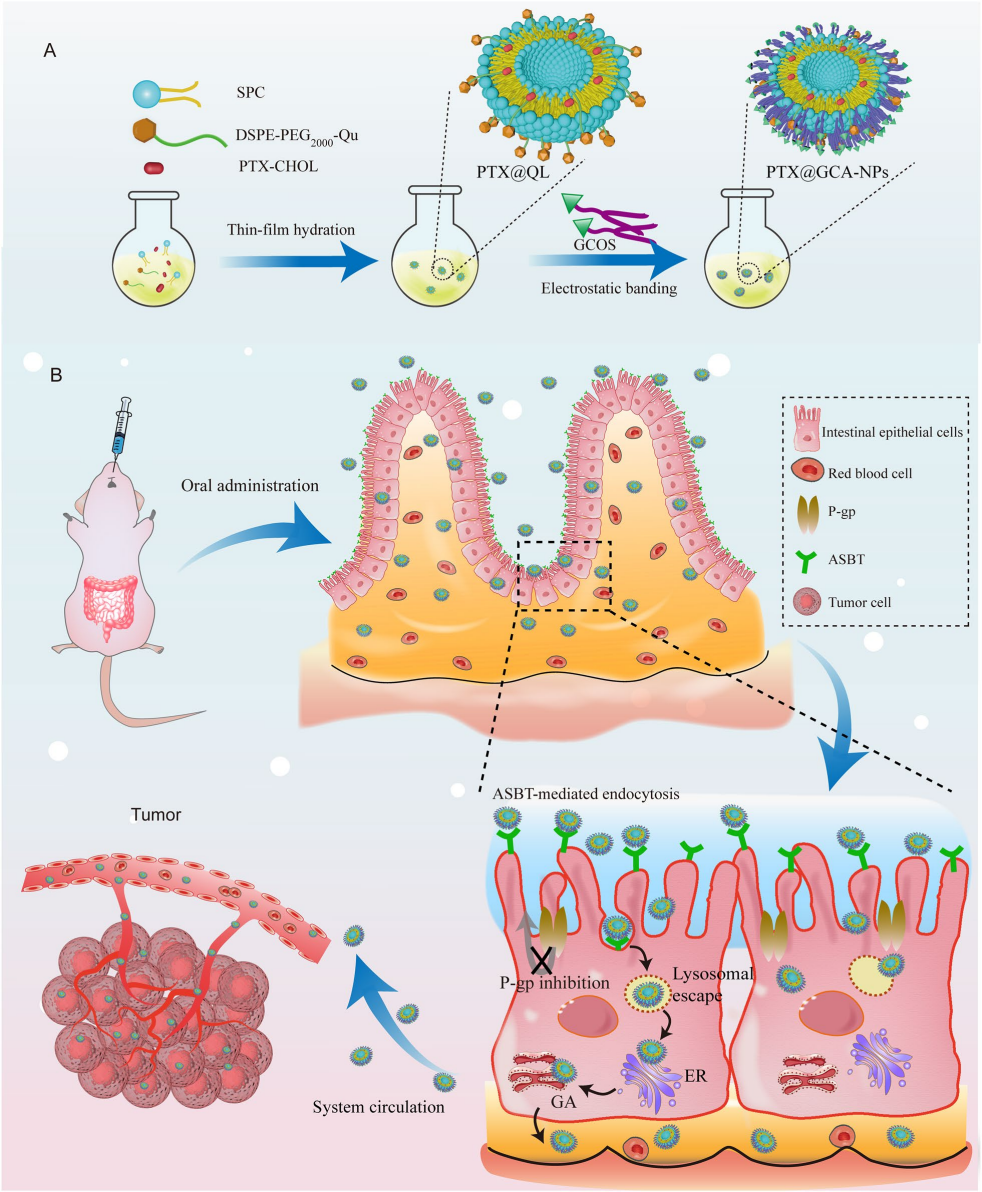
Schematic illustration. **A** Preparation of oral PTX@GCA-NPs delivery system **B** Process of intestinal enterocyte transportation of PTX@GCA-NPs (ER: endoplasmic reticulum; GA: Golgi complex apparatus)

Herein, a PTX-cholesterol complex (PTX-CHOL) was synthesized and encapsulated in GCA-NPs to achieve enhanced stability [36]. As illustrated in Fig. 1, the synthesized materials GCOS could coated on the surface of PTX-loaded liposomes, thereby tightening the liposomes core and showing active intestinal targeting. After orally administration, the nanoparticles would undergo ASBT-mediated transcellular transport. This study discussed the properties of GCA-NPs in detail, including stability in the GIT environment, endocytosis and transport behavior in Caco-2 monolayers, colocalization with ASBT in cell monolayers and villi of enterocytes, retention effect in the intestine, oral bioavailability, and antitumor effect in vivo. Our novel oral delivery system showed remarkable efficacy in overcoming multiple barriers and achieving superior antitumor efficacy compared with intravenously administered with Taxol^®^, demonstrating that GCA-NPs can be used for oral chemotherapy. Moreover, GCA-NPs can be used as promising carriers for oral delivery of more extensive drugs, such as peptides, and can improve the treatment of other diseases.

## Results and discussion

### Synthesis and characterization of GCOS and DQ

**Fig. 2:**
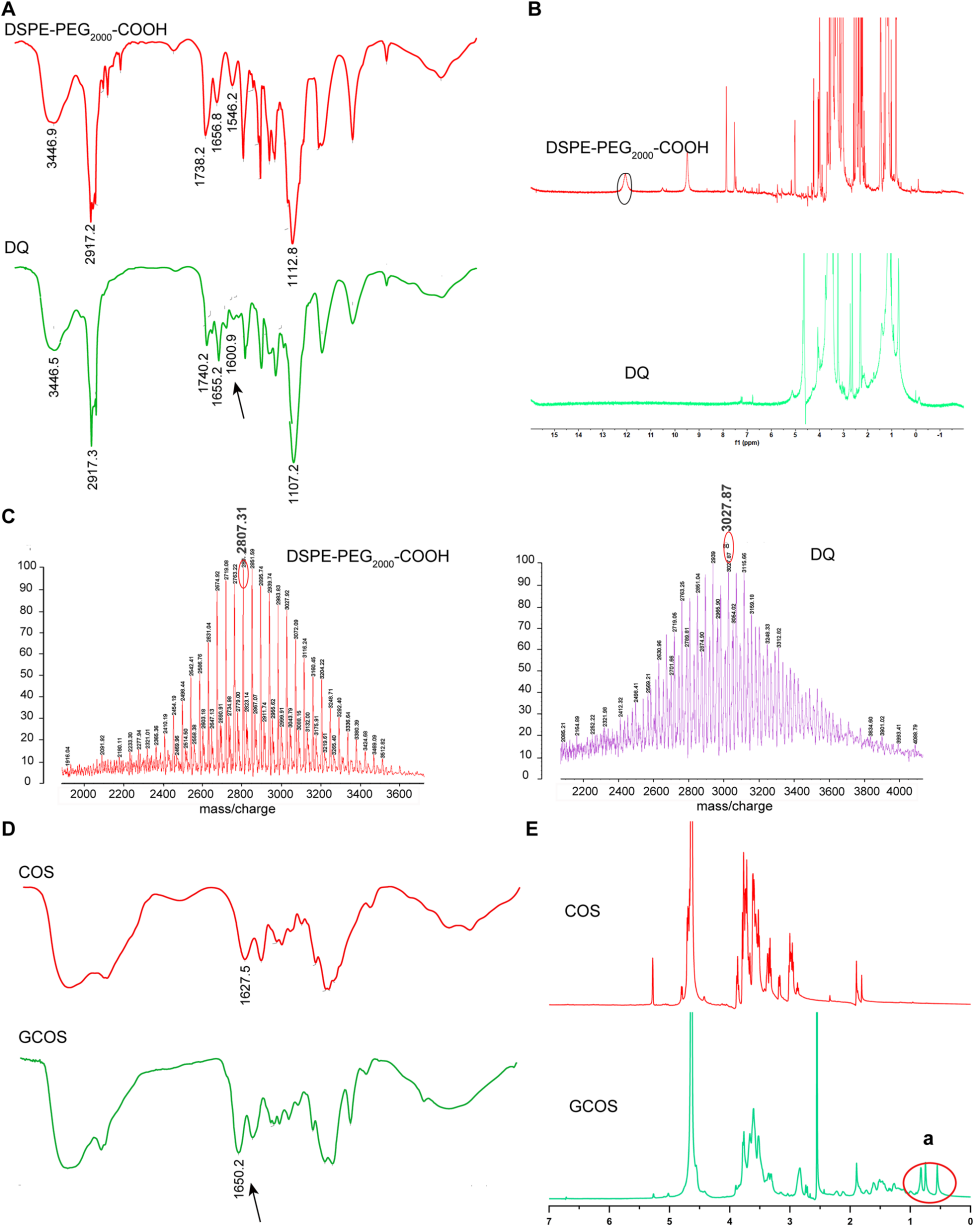
Preparation and characterization of DSPE-PEG_2000_-modified quercetin (DQ) and GCA-modified COS (GCOS). **A** FT-IR spectrum of DSPE-PEG_2000_-COOH and DQ. **B** Characteristic peaks of DSPE-PEG_2000_-COOH and DQ by ^1^H-NMR. **C** MALDI-TOF spectrum of DSPE-PEG_2000_-COOH and DQ. **D** FT-IR spectrum of COS and GCOS. **E** Characteristic peaks of COS and GCOS by ^1^H-NMR. **a** is corresponding to the protons of GCA residues

GCOS and DQ were synthesized according to the process shown in Additional file 1: Fig. S1. Qu, a natural flavonoid compound, competitively inhibits members of the muti-drug resistance (MDR) family. Previous studies have reported that Qu can be a potential P-gp inhibitor [24, 25]. Therefore, in this study, Qu was selected as a P-gp inhibitor to decorate DSPE-PEG_2000_-COOH to obtain a novel material, DQ. The chemical structure was confirmed by FT-IR, ^1^H-NMR, and MALDI-TOF (Fig. 2A-C). Broadly, the peak at 1600.9 cm^−1^ corresponded to the characteristic peak of the aromatic ring of Qu (Fig. 2A). The characteristic peak of the carboxyl group of DSPE-PEG_2000_-COOH disappeared at 12–13 ppm owing to conjugation between the carboxyl and hydroxyl groups (Fig. 2B). The molecular weight characterized by MALDI-TOF was increased from 2807.31 to 3027.87 Da (Fig. 2C), proving that Qu was successfully conjugated with DSPE-PEG_2000_-COOH.

COS is a positively charged natural polysaccharide with excellent mucoadhesive properties, biodegradability, and biocompatibility. Compared to chitosan, COS has a low molecular weight and good hydrophilicity. These properties make COS as an ideal material for nanocarrier drug delivery. In this study, we conjugated GCA with COS via an amidation reaction between the amino groups of COS and carboxyl groups of GCA to obtain GCOS. The chemical structure was verified by ^1^H NMR and FT-IR spectroscopy. The increase at 1627.5 cm^−1^ in the IR spectrum indicated the formation of an amide bond (Fig. 2D). In addition, the characteristic peak corresponding to GCA appeared at 0–1 ppm of GCOS, confirming the successful conjugation of COS with GCA (Fig. 2E). MALDI-TOF spectrum showed that after decoration of GCA, the molecular weight showed increase from 1580.86 Da to 2027.6 Da (Additional file 1: Fig. S2). Indicating that we have successfully synthesized GCOS.

### Synthesis of PTX-cholesterol complex

According to a previous study [37, 38], PTX-loaded lipid nano-emulsions were stable for only 8 days at 4 ℃ owing to the poor lipophilicity of PTX. Therefore, we prepared a PTX-CHOL complex with stronger lipophilicity to form PTX-loaded liposomes. PTX-CHOL was prepared as previously described [38]. Differential scanning calorimetry (DSC) was used to verify the interaction between PTX and cholesterol in the PTX-CHOL complex. The DSC curves for PTX, cholesterol, and PTX-CHOL are shown in Additional file 1: Fig. S3. Melting absorption peaks of cholesterol (Additional file 1: Fig. S3A) and PTX (Additional file 1: Fig. S3B) were at 148.6 ℃ and 226.7 ℃, respectively. A new melting absorption peak (138.7 ℃) appeared in the PTX-CHOL DSC result (Additional file 1: Fig. S3C), whereas the PTX and cholesterol peaks disappeared, indicating that the pure PTX-CHOL complex was successfully synthesized.

### Preparation and characterization of nanoparticles

**Fig. 3:**
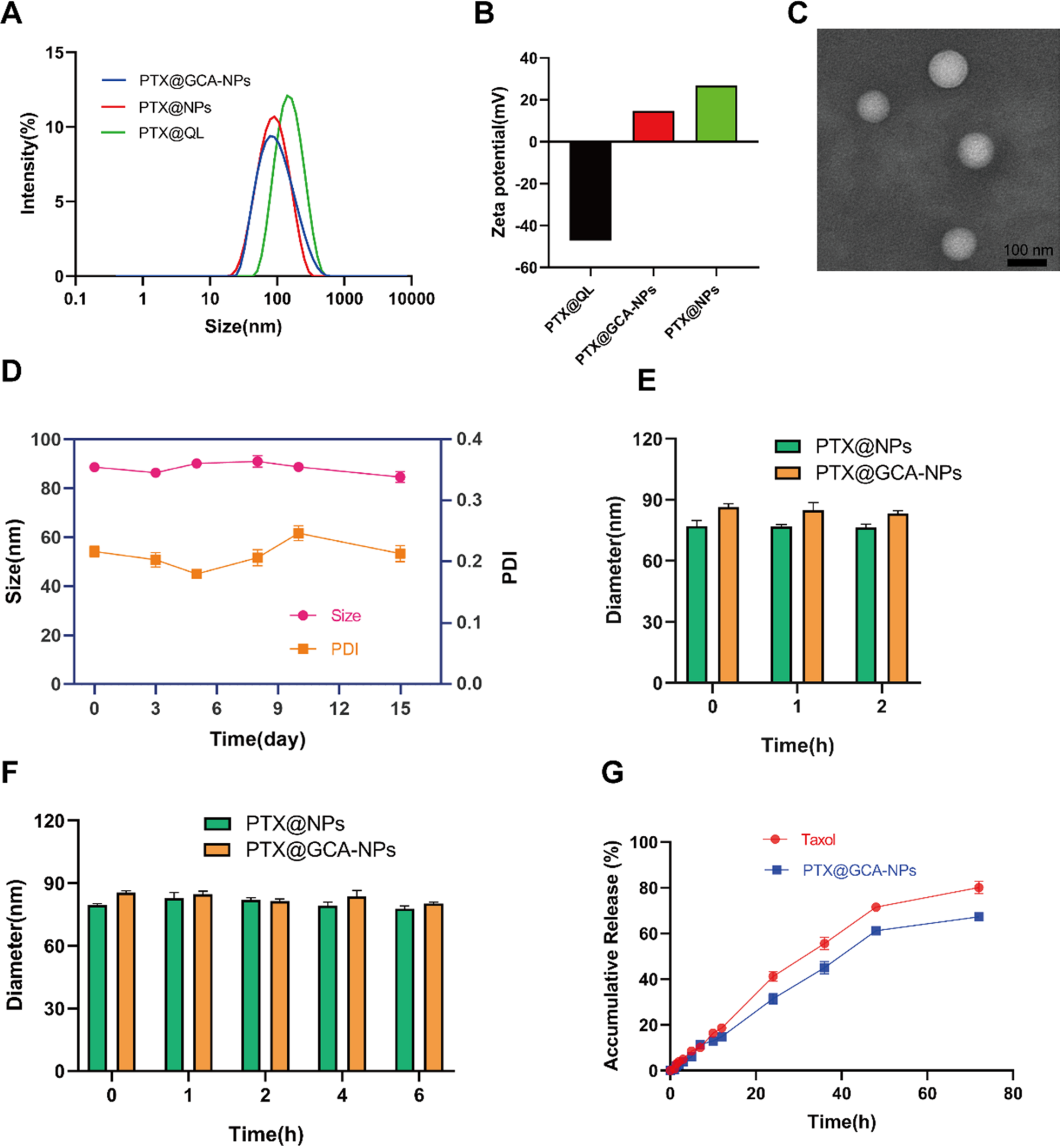
Characterization of nanoparticles. **A** Average diameter of PTX@GCA-NPs (blue), PTX@NPs (red), PTX@QL (green). **B** Zeta potential of PTX@GCA-NPs, PTX@NPs and PTX@QL. **C** TEM images of PTX@GCA-NPs (scale bar: 100 nm). **D** Storage stability of PTX@GCA-NPs in 4 ℃. **E** Average diameter of PTX@GCA-NPs in SGF. **F** Average diameter of PTX@GCA-NPs in SIF. **G** Drug release behavior in SGF and SIF conditions, the first 2 h was in SGF medium and the followed 70 h was in SIF medium. Data is presented as mean ± SEM, n = 3

The ASBT-mediated PTX-loaded oral delivery system was obtained in two steps. In the first step, PTX-encapsulating Qu-modified liposomes (PTX@QL) were prepared using the thin-film hydration method, as previously described [37]. Briefly, the major components PTX-CHOL, DQ, soybean phospholipid (SPC), DSPE-mPEG_2000_, and cholesterol tend to form vesicles due to van der Waals interaction forces. After hydration, we obtained a stable liposome (PTX@QL) containing both PTX (with antitumor activity) and Qu (with P-gp inhibition effect). Dynamic light scattering (DLS) results showed that the average size and zeta potential of PTX@QL were approximately 118.5 nm and − 44.7 mV, respectively, and the encapsulation and loading rates were 98% and 2%, respectively (Fig. 3A, B, Additional file 1: Table S1). In the second step, GCOS-coated PTX-loaded QL (PTX@GCA-NPs) was prepared by making use of the electrostatic interactions between the positively charged GCOS and negatively charged PTX@QL. The average size of the PTX@GCA-NPs was reduced to 87.5 nm, while the zeta potential increased to + 14.1 mV. The inversion of the surface charge confirmed the successful synthesis of the GCOS-coated nanoparticles. The reduced particle size indicated the good stability of the prepared delivery system. Transmission electron microscopy (TME) images of the nanoparticles displayed a spherical structure with a diameter of approximately 80 nm (Fig. 3C), consistent with the DLS particle size results (Additional file 1: Table S1). The storage stability test showed that PTX-loaded GCA-NPs were stable for 15 days at 4 °C (Fig. 3D). PTX@NPs were prepared with COS instead of GCOS, using the same method. Nanoparticles without Qu were named PTX@GLNPs. Both PTX@NPs and PTX@GLNPs were used as controls.

After oral administration, nanoparticles experience a complex gastrointestinal environment of low pH containing various enzymes. Therefore, gastrointestinal stability is essential for oral nanoparticles to retain their efficacy. The stability of PTX@GCA-NPs in simulated gastric fluid (SGF) and simulated intestinal fluid (SIF) is shown in Fig. 3E, F. There was no significant change in particle size after incubation in SGF for 2 h. Furthermore, the size of PTX@GCA-NPs was stable after incubation in SIF for 6 h. The above results demonstrate that PTX-loaded GCA-NPs were able to resist the destruction of the nanoparticle structure in the gastrointestinal environment and were absorbed in the small intestine with a relatively complete structure.

### In vitro drug-release behavior in SGF and SIF

To further study the stability of PTX@GCA-NPs in SGF and SIF, the drug release profiles of PTX-loaded GCA-NPs were investigated in vitro in SGF and SIF. The PTX-loaded GCA-NPs were stable in SGF for 2 h (Fig. 3G); the cumulative release was less than 3%, which was slightly lower than that of Taxol^®^ (approximately 4%). After transferring into SIF and incubation for another 70 h, the PTX-loaded GCA-NPs exhibited controlled-release behavior. Notably, the cumulative release was less than 15% after incubation for 10 h, demonstrating the remarkable stability of the PTX-loaded GCA-NPs in a simulated gastrointestinal environment. These results indicated that PTX-loaded GCA-NPs were expected to resist acidic and enzymatic degradation in the stomach and intestine, ensuring intestinal uptake of intact nanoparticles.

### Cytotoxic analysis of PTX@GCA-NPs

To be useful, at the appropriate concentration, oral preparations should be biocompatible and should not affect the biological functions of intestinal epithelial cells. Therefore, the cytotoxicity of blank GCA-NPs and PTX@GCA-NPs was evaluated using a CCK-8 assay in Caco-2 cells. As is shown in Additional file 1: Fig. S4A, after 24 h of incubation, the optimal survival rate of Caco-2 cells was maintained in all groups, demonstrating that blank GCA-NPs exhibited good biocompatibility with intestinal cells. Similarly, when Caco-2 cells were incubated with various concentrations of PTX@GCA-NPs equivalent to 1–200 μg/mL PTX for 4 h, all groups exhibited low cytotoxicity with cell viability of more than 80% (Additional file 1: Fig. S4B). The results indicate that PTX@GCA-NPs at predetermined concentrations and incubation times did not significantly affect the biological activity of Caco-2 cells.

### Endocytosis and ASBT-mediated endocytosis pathway studies of GCA-NPs

**Fig. 4:**
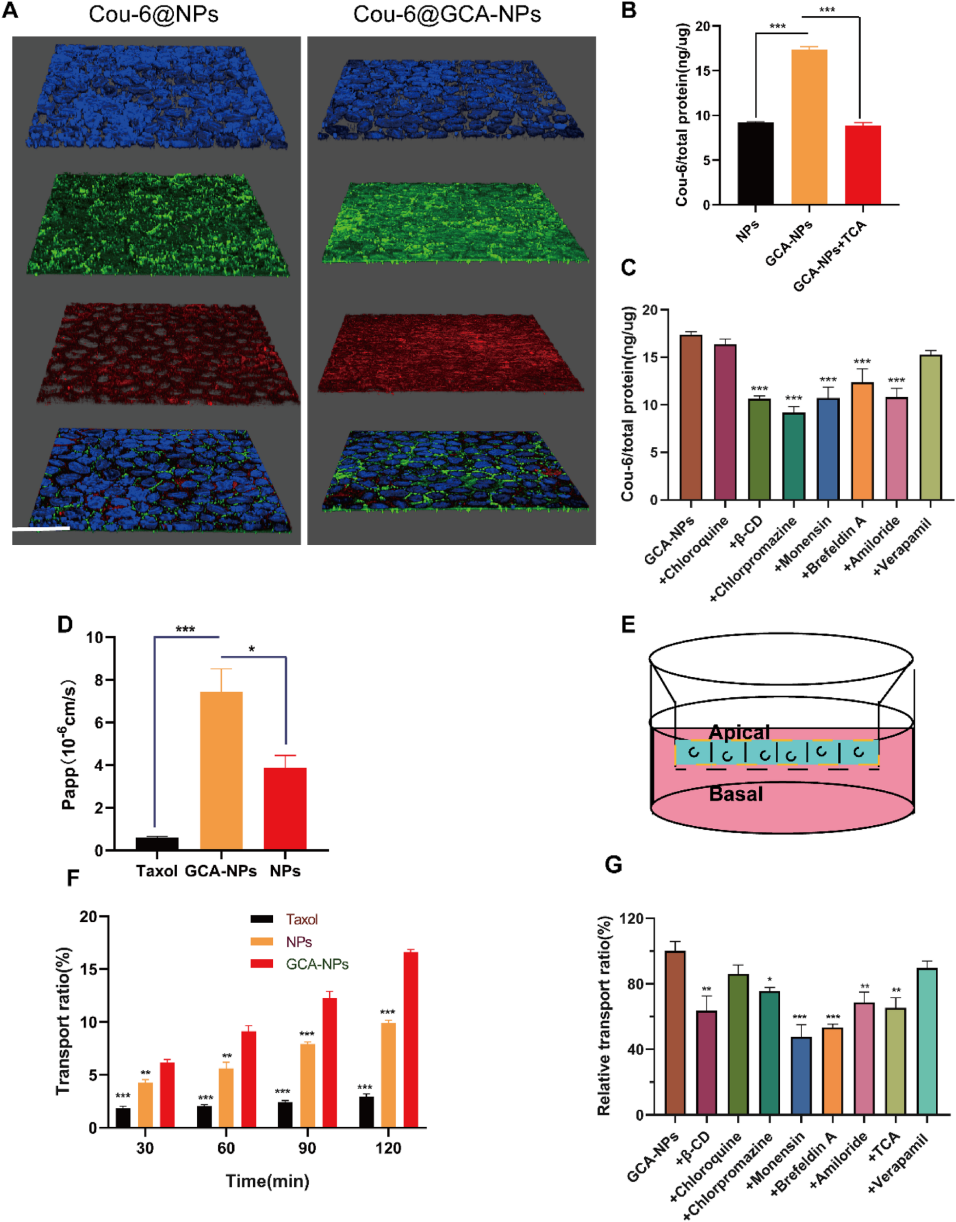
Endocytosis and transport mechanism of GCA-NPs in Caco-2 cell monolayers. **A** 3D images of Caco-2 cells monolayers after incubated for Cou-6@GCA-NPs and Cou-6@NPs for 1 h. Cell nucleus were labeled with DAPI (blue), ASBT were stained with anti-SLC10A2 rabbit polyclonal antibody and labeled with Alexa Fluor® 647 labeled goat anti-rabbit secondary IgG (red). (scale bar: 25 μm, magnification: 630 ×). **B** Cellular uptake of Cou-6@GCA-NPs in monolayers after treated with GCA-NPs, NPs, GCA-NPs + M-β-CD (β-CD), GCA-NPs + chloroquine (Chloroquine), GCA-NPs + chlorpromazine (Chlorpromazine), GCA-NPs + monensin (Monensin), GCA-NPs + brefeldin A (brefeldin A), GCA-NPs + amiloride (Amirolide), GCA-NPs + TCA (TCA), GCA-NPs + verapamil (Verapamil). **C** Relative uptake ratio of Cou-6@GCA-NPs in monolayers. **D** Apparent permeability efficiency (Papp) of Taxol^®^, PTX@GCA-NPs and PTX@NPs when penetrated through monolayers after 2 h. **E** Transwell model. **F** Transport ratio of Taxol^®^, PTX@GCA-NPs and PTX@NPs in predetermined time points (30 min, 60 min, 90 min, 120 min) in the process of penetrating Caco-2 monolayers. **G** Relative transport ratio of GCA-NPs (control) after 2 h, when pretreated with various inhibitors including M-β-CD, chloroquine, chlorpromazine, monensin, brefeldin A, amiloride, TCA, verapamil. Data is presented as mean ± SEM, n = 3, ***P < 0.001, **P < 0.01, *P < 0.05

To investigate the cellular uptake efficiency of GCA-NPs in intestinal epithelial cells, Caco-2 monolayers were used for endocytosis studies. According to a previous study by Fan, Caco-2 cell monolayers differentiate into intestinal epithelial structures and overexpress ASBT after three weeks of cultivation [20]. Therefore, Caco-2 cell monolayers are ideal models for in vitro studies. We used a coumarin-6 (Cou-6) fluorescent dye and confocal laser spectrum microscopy (CLSM) to observe ASBT-mediated cellular uptake. After the formation of Caco-2 cell monolayers, ASBT was labeled with an anti-SLC10A2 primary antibody and detected using Alexa Fluor^®^ 647 conjugated secondary antibodies. As shown in Fig. 4A, ASBT was uniformly distributed in monolayers. Notably, Cou-6@GCA-NPs showed stronger fluorescence intensity than Cou-6@NPs in the monolayers.

Regarding cellular uptake efficiency, as shown in Fig. 4B, the Cou-6@GCA-NPs group exhibited higher internalization efficiency than the Cou-6@NPs group. Moreover, compared with Cou-6@NPs, Cou-6@GCA-NPs showed a 1.87-fold increase in the amount of Cou-6 internalized into Caco-2 monolayers after incubation for 1 h, suggesting that GCA functionalization contributed to the enhanced endocytosis of nanoparticles via the ASBT-mediated pathway. To further study the role of ASBT in enhancing cellular uptake, we added 100 μM taurocholic acid (TCA) to cell monolayers to competitively inhibit bile-acid transporters [39]. Consistently, we found that endocytosis efficiency decreased by approximately twofold compared with that in monolayers not treated with TCA.

This study also investigated other endocytosis pathways of Cou-6@GCA-NPs using Caco-2 cell monolayers. As shown in Fig. 4C, various inhibitors, including methyl-β-cyclodextrin (M-β-CD, an inhibitor of lipid rafts), chloroquine (an inhibitor of lysosomes), chlorpromazine (an inhibitor of clathrin-mediated endocytosis), monensin (an inhibitor of the Golgi complex-basolateral membrane pathway), brefeldin A (an inhibitor of the ER-Golgi complex transport pathway), amiloride (a micropinocytosis inhibitor), and verapamil (a P-gp inhibitor), were added separately to the monolayers before treatment with preparations loaded with Cou-6. Besides ASBT-mediated cellular uptake, lipid rafts and the clathrin pathway also showed significant effects on the process of endocytosis of GCA-NPs. Notably, after pretreatment of the cell monolayers with chloroquine for 1 h, no obvious decrease in endocytosis efficiency was observed compared to the untreated group. Chloroquine was used as an inhibitor of lysosomes because it can inhibit the activity of enzymes and prevent acidification of lysosomes [20]. We speculated that the GCA-NPs might have resisted degradation in lysosomes owing to their positive charge.

P-gp-mediated efflux is a major barrier not only in intestinal absorption but also in absorption in the tumor environment. Hence, we co-delivered Qu to enhance the oral bioavailability and antitumor effects of PTX [32]. Verapamil is a P-gp inhibitor; however, no evident change in uptake by Caco-2 monolayers was observed with or without verapamil pretreatment (Fig. 4C). These results indicate that the novel oral nano-delivery systems can inhibit the P-gp efflux pump.

### Transepithelial transport studies using Caco-2 cell monolayers

When orally administered nanoparticles enter the small intestine, crossing the intestinal epithelial barrier is the limiting factor for drug efficacy [40]. Before reaching blood circulation, oral drugs must permeate through the intestinal epithelium [41]. We analyzed the trans-epithelial efficiency of GCA-NPs in Caco-2 cell monolayers. Chitosan has been reported to open the tight junctions of cell monolayers and increase drug permeability. Hence, after treatment with Taxol^®^, PTX@NPs, and PTX@GCA-NPs, we recorded the transepithelial electrical resistance (TEER) of Caco-2 cell monolayers to monitor their integrity. No obvious decrease was observed in the TEER value, indicating that the above preparations did not affect the biological activity and integrity of Caco-2 cell monolayers after treatment for 3 h (Additional file 1: Fig. S5). During intestinal absorption, oral preparations contact the apical membranes of the intestinal epithelium, transport into the cytoplasm after permeation through the mucus layer, and finally undergo exocytosis at the basolateral membrane [8]. Therefore, we added these preparations to the apical side of the cell monolayers and sampled a certain quantity of solution from the basolateral side at predetermined time points.

To explore the ability of PTX@GCA-NPs to enhance transepithelial permeability, we incubated monolayers with Taxol^®^, PTX@NPs, and PTX@GCA-NPs for 2 h, and analyzed the PTX content in the samples collected from the basolateral side using HPLC. As shown in Fig. 4D, the apparent permeability coefficient (Papp) value of PTX@GCA-NPs was approximately 7.44 × 10^−6^ cm/s, which was 1.9- and 12.5-fold higher than that of PTX@NPs and Taxol^®^, respectively, indicating a higher permeability of PTX@GCA-NPs across the intestinal barrier. Although the Papp value of PTX in each group was quite low because of the poor permeability of PTX, PTX@GCA-NPs showed an obviously enhanced Papp value compared to PTX@NPs and Taxol^®^ groups. The transepithelial transport of PTX exhibited a time-dependent behavior (Fig. 4F). The transport ratio of the PTX@GCA-NPs groups reached approximately 16.6% after treatment with monolayers for 2 h, which was 1.7- and 5.6-fold higher than that of PTX@NPs and Taxol^®^, respectively. The transepithelial permeability of PTX was significantly enhanced after encapsulation in GCA-modified COS-coated nanoparticles, indicating that orally administered GCA-NPs have the potential to overcome the intestinal epithelial barrier.

Trranscellular pathway plays important role in epithelial transportation [42]. To further illustrate the transepithelial pathway of GCA-NPs, various inhibitors, including M-β-CD, chloroquine, chlorpromazine, monensin, brefeldin A, amiloride, TCA, and verapamil, were investigated in accordance with endocytosis pathway studies. It was observed that ASBT, lipid rafts, the micropinocytosis-mediated pathway, and particularly the ER-Golgi complex-membrane process, played an important role in transepithelial transport of GCA-NPs (Fig. 4G). When oral nanoparticles are endocytosed into the cytoplasm, they usually interact with a series of organelles. First, oral nanoparticles escape lysosomes and they are consequently then transported into the ER and Golgi complexes [40]. Brefeldin A and monensin block the ER-Golgi complex and Golgi complex-basolateral membrane transport pathways, respectively. Notably, after pre-incubation with brefeldin A and monensin for 1 h, the transport efficiency of Cou-6@GCA-NPs in Caco-2 monolayers decreased by 3.7-and 4.2-fold, respectively, demonstrating that the ER-Golgi-membrane pathway participated in the cytoplasmic trans-endocytosis process. Considering that drugs might be degraded by abundant enzymes in lysosomes, it is prudent to design nanoparticles that are capable of quickly escaping lysosomes. Encouragingly, no decrease in the apparent transport ratio was observed when monolayers were pre-incubated with chloroquine, suggesting that GCA-NPs were able to achieve endosomal escape and protect the encapsulated drugs from degradation. Verapamil is a classic P-gp inhibitor; however, the transport ratio of GCA-NPs was similar in monolayers with or without verapamil pretreatment (Fig. 4G), in accordance with the results of endocytosis pathway studies. Based on the above data, we conclude that GCA-NPs have the potential to overcome the barriers of P-gp pump efflux and endosomal degradation. In addition, the ER and Golgi complexes play a dominant role in GCA-NPs cellular transport.

### FRET analysis

Förster resonance energy transfer (FRET) technology was used to study the integrity of the nanoparticles. A FRET pair, DiO and DiI, was co-loaded into DiO/DiI@GCA-NPs. DiO was the donor and DiI was the acceptor. When the distance between DiO and DiI is no more than 10 nm, the energy of DiO emission can excite DiI under the excitation wavelength of DiO, showing an increased signal at 565 nm and a decreased signal at 501 nm [43]. As displayed in Additional file 1: Fig. S6A, the fluorescence spectrum of DiO/DiI@GCA-NPs in water showed a strong FRET signal, with peaks at 501 and 565 nm and a FRET ratio of 54%. After mixing with acetone to destroy the structure of the nanoparticles, the FRET ratio decreased significantly to 21% (Additional file 1: Fig. S6B), demonstrating that disruption of the nanoparticle structure had occurred. Moreover, compared with DiO@GCA-NPs and DiI@GCA-NPs, an obvious increase in the fluorescence intensity at 565 nm was observed when DiO and DiI were encapsulated into GCA-NPs simultaneously, indicating the occurrence of FRET (Additional file 1: Fig. S6C). The above experiments indicated that FRET technology can be used to measure nanoparticle integrity.

**Fig. 5:**
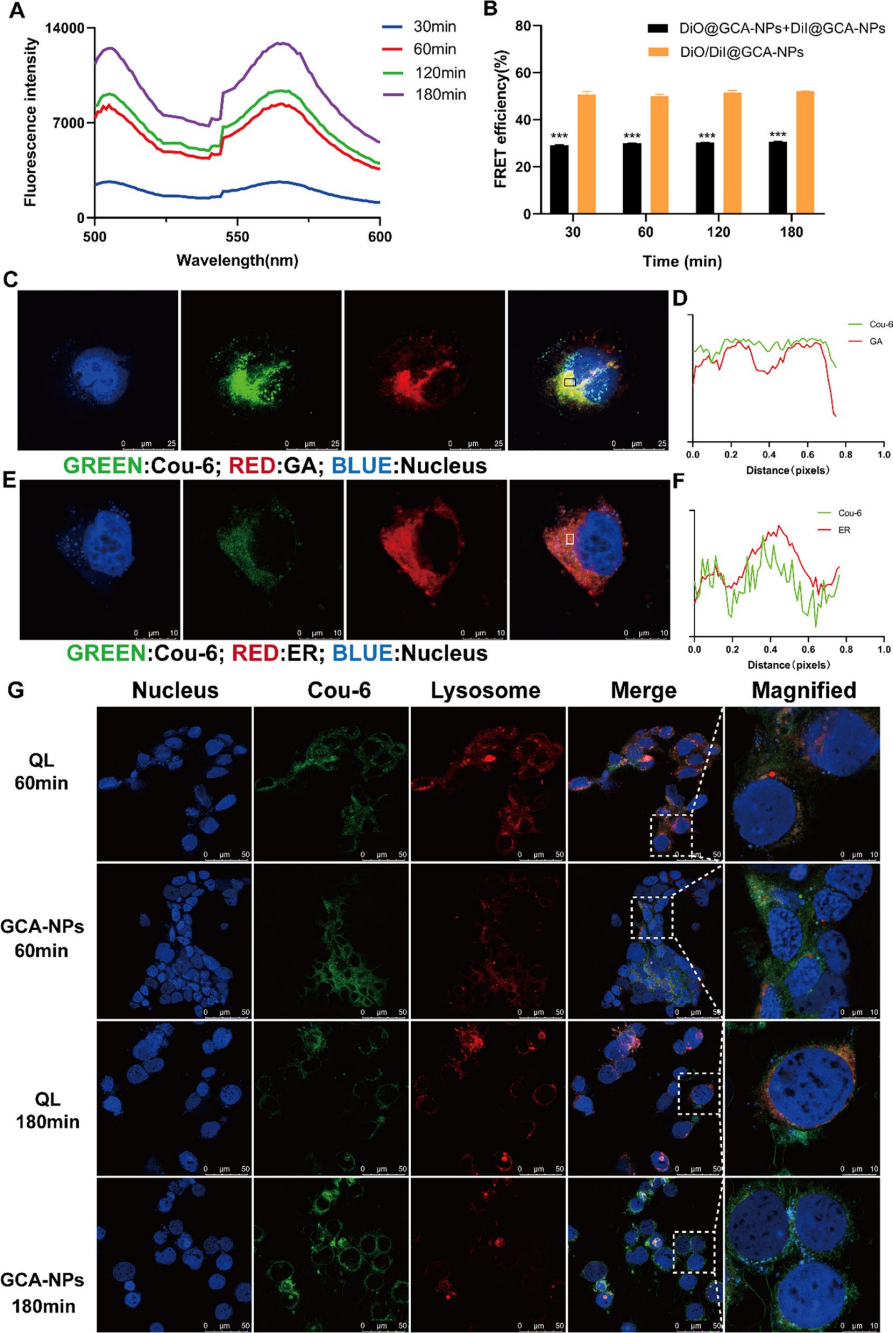
Cellular trafficking pathway of GCA-NPs. **A** The emission spectrum of basolateral medium was assayed after incubated with DiO/DiI@GCA-NPs for 30 min, 60 min, 120 min, 180 min. **B** FRET efficiency of DiO/DiI GCA-NPs and DiO@GCA-NPs + DiI@GCA-NPs after transport across Caco-2 cell monolayers for 30 min, 60 min, 120 min, 180 min. The excitation wavelength was 480 nm. **C** Colocalization of Cou-6@GCA-NPs and Golgi apparatus (GA). Cell nucleus was stained with DAPI (blue) and GA complex was labeled with GA tracker (red). (scale bar: 25 μm, magnification: 630 ×). **D** Colocalization analysis of Cou-6@GCA-NPs and GA. **E** Colocalization of Cou-6@GCA-NPs and endoplasmic reticulum (ER). Cell nucleus was stained with DAPI (blue) and ER were labeled with ER tracker (red). (scale bar: 10 μm, magnification: 630 ×). **F** Colocalization analysis of Cou-6@GCA-NPs and ER. **G** Cellular colocalization of Cou-6@GCA-NPs and Cou-6@QL with lysosome at 60 min and 180 min, respectively. Cell nucleus was labeled with DAPI (blue) and lysosomes were stained with lysotracker (red). (scale bar: 10 μm, magnification: 630 ×). Data is presented as mean ± SEM, n = 3, ***P < 0.001

To study the integrity of GCA-NPs penetrating Caco-2 cell monolayers, DiO/DiI@GCA-NPs and DiO@GCA-NPs + DiI@GCA-NPs mixtures were incubated with the monolayers. At regular time points, the medium on the basal side was collected and absorbance was measured using a microplate reader. Figure 5A showed a strong FRET phenomenon with two peaks at 501 and 565 nm at different time points. The increase in the fluorescence intensity over time resulted from the accumulation of dyes transported across the monolayers. In addition, we calculated the FRET ratio of the DiO/DiI@GCA-NPs and DiO@GCA-NPs + DiI@GCA-NPs mixture groups. Notably, the FRET ratio of DiO/DiI@GCA-NPs at different time points (30, 60, 120, and 180 min) was greater than 50%, which was higher than that observed for the mixture group (30%) (Fig. 5B and Additional file 1: Table S2). Meanwhile, no obvious change in the FRET ratio occurred with the extension of transportation time. Based on these results, we concluded that GCA-NPs were capable of maintaining an intact structure during penetration across intestinal epithelial cells.

### Visualization of intracellular trafficking of Cou-6@GCA-NPs

Based on the results of the transepithelial pathway studies, we speculated that the ER and Golgi complex participate in the intracellular trafficking of GCA-NPs. In addition, as no obvious change in the transport ratio was observed when monolayers were pre-incubated with the lysosome inhibitor chloroquine, we concluded that GCA-NPs are capable of achieving endosomal escape in the process of intracellular trafficking. The intracellular transport route was visualized by CLSM using colocalization analysis of Cou-6@GCA-NPs with lysosomes, ER, and Golgi complexes.

ER and Golgi complexes overlapped with Cou-6@GCA-NPs after incubation for 2 h (Fig. 5C–F), demonstrating that GCA-NPs accumulated in the ER and Golgi complexes after endocytosis into Caco-2 cells. The colocalization fluorescence signal between Cou-6@GCA-NPs and lysosomes was observed clearly at 60 min post-incubation, suggesting that GCA-NPs were primarily entrapped into endosomes/lysosomes (Fig. 5G). However, after 3 h of incubation, the fluorescence signals of GCA-NPs separated from the lysosomes, whereas most of the QL remained trapped in the lysosomes. In addition, the fluorescence intensity of the lysosomal signal of the Cou-6@GCA-NPs groups was weakened, which might be attributed to lysosomal cleavage. These results indicate that GCOS modification plays a critical role in the lysosomal escape of GCA-NPs.

### Biodistribution, retention, and permeability of nanoparticles in the intestine

**Fig. 6:**
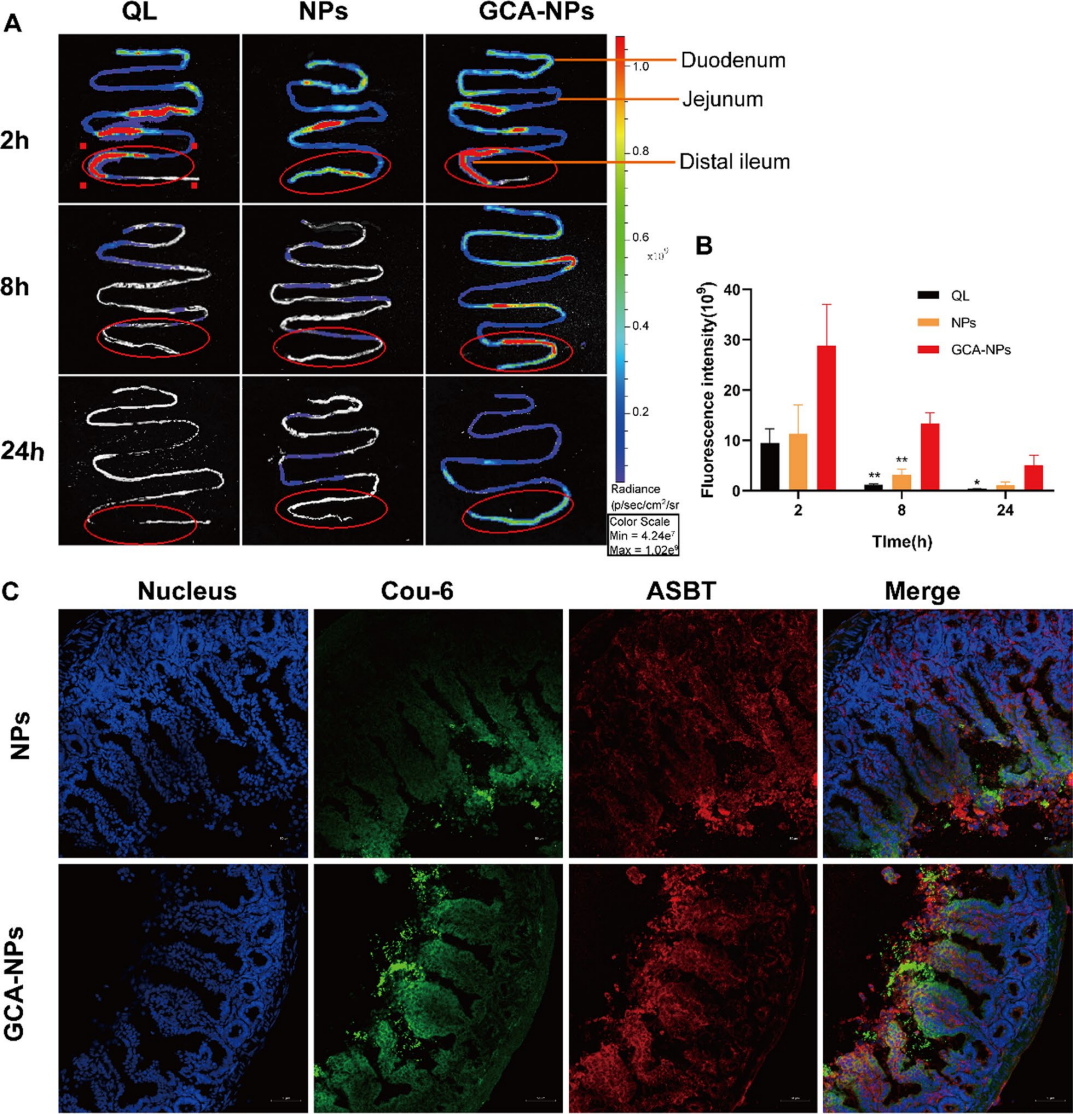
Intestinal and ileum’s enterocytes biodistribution studies. **A** Intestinal biodistribution after mice was orally administered with 2.5 mg/kg of DiR@QLs, DiR@NPs and DiR@GCA-NPs for 2 h, 8 h, 24 h, respectively. **B** Fluorescence intensity of selected area in ileum. **C** Visualization of absorption effects of Cou-6@NPs and Cou-6@GCA-NPs in distal ileum after gavage for 2 h. (scale bar: 50 μm, magnification: 200 ×). Data is presented as mean ± SEM, n = 3, **P < 0.01, *P < 0.05

The in vivo real-time biodistribution in the intestine was investigated after the oral administration of DiR@GCA-NPs, DiR@NPs, and DiR@QL at predetermined time points, and the intestine was removed to analyze the fluorescence signal. According to a previous study, ASBT showed remarkable expression in the distal ileum; therefore, bile acid-modified nanoparticles were expected to have enhanced permeability in the distal ileum [18]. Figure 6A shows the preferred residence of DiR@GCA-NPs in the distal ileum at different time points compared with other intestinal segments. Consistently, the DiR@GCA-NP group showed stronger signals than the other two groups (NPs and QL). Moreover, at 24 h post-administration, the fluorescence intensity of the DiR@GCA-NPs group was still marked in the distal ileum, compared to the DiR@NPs and DiR@QL groups. Similarly, the tendency of the fluorescence signal in the isolated distal ileum segment 24 h after administration was consistent with the in vivo real-time biodistribution. As shown in Fig. 6B, the fluorescence intensity of the GCA-NPs group in the distal ileum segment was 2.8-fold and 2.4-fold higher than that of the NPs and QL groups, respectively. These results indicate that GCA-NPs can improve the intestinal retention time and prolong the absorption time, which was attributed to the mucoadhesive properties of COS.

To further investigate the permeation of GCA-NPs and NPs into the villi of the distal ileum, the oral absorption of nanoparticles was visualized using CLSM. BABL/c nude mice were orally administered 10 mg/kg of Cou-6@GCA-NPs or Cou-6@NPs, and 2 cm of the distal ileum was subsequently collected and sliced. The ASBT protein expressed in the ileum was labeled with anti-SLC10A2 primary antibodies and detected using Alexa Fluor® 647 conjugated secondary antibodies, which are represented by a red signal in the images. As can be seen in Fig. 6C, the red signal is the ASBT protein, while the green signal represents the nanoparticles. The fluorescence intensity of Cou-6@GCA-NPs in the ileum microvilli was higher than that of Cou-6@NPs. We speculated that GCA-NPs exhibited high permeability across the ileum via the ASBT-mediated pathway, which may contribute to enhancing the oral bioavailability of poorly permeable drugs.

### In vivo pharmacokinetics

**Fig. 7:**
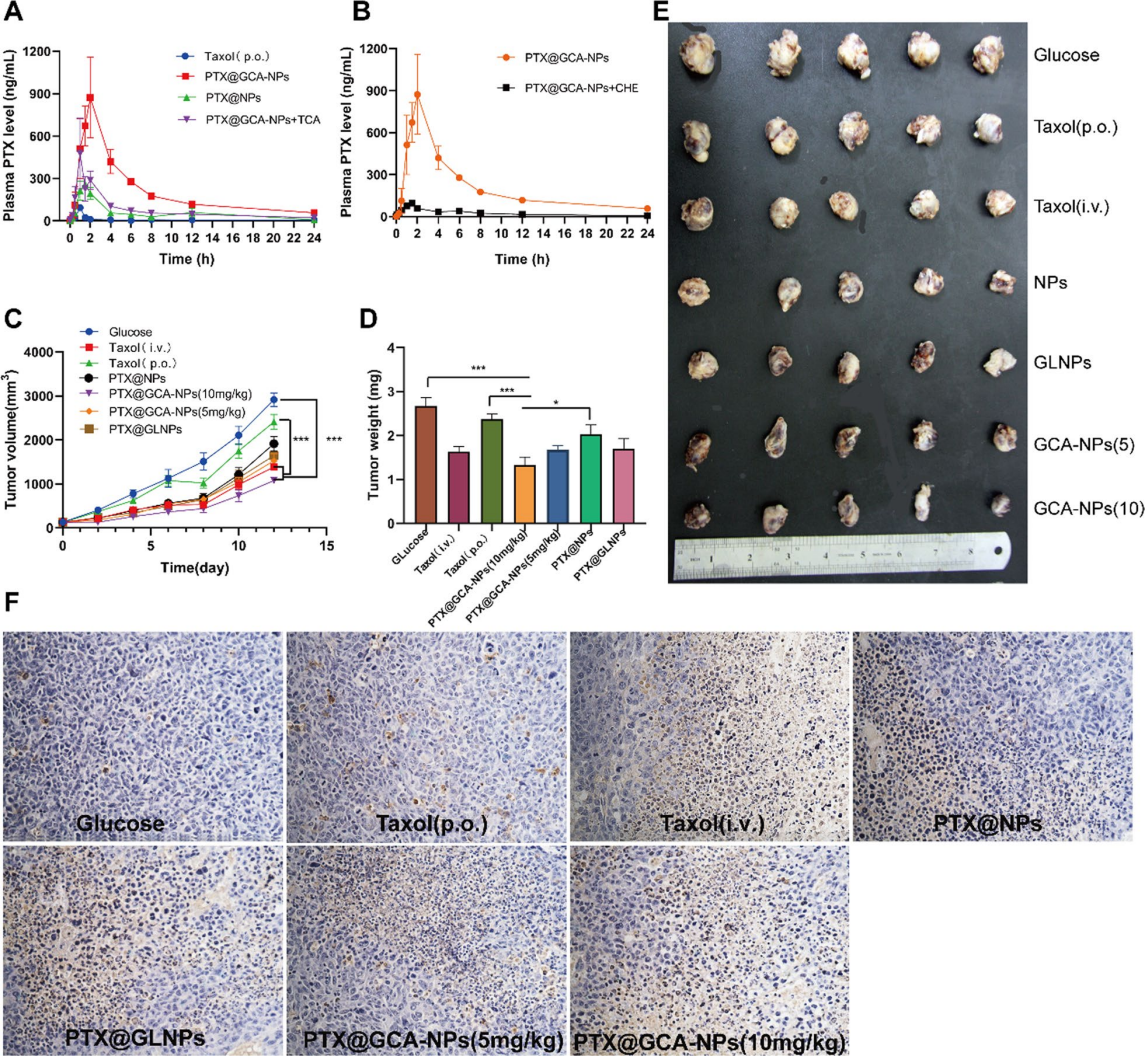
In vivo pharmacokinetics and anti-tumor efficacy. Plasma drug curves after oral administration of **A** Taxol^®^, PTX@NPs, PTX@GLNPs, PTX@GCA-NPs, PTX@GCA-NPs + TCA (p.o.). **B** PTX@GCA-NPs, PTX@GCA-NPs + CHE (s.c.) at a PTX dose of 10 mg/kg. n = 3. **C** The changes of tumor volume after treatments. **D** Tumor weights. **E** Tumor photos at days 19 post-inoculation. GCA-NPs (5) and GCA-NPs (10) refer to 5 mg/kg and 10 mg/kg dosage of PTX, respectively. **F** Representative images of TUNEL assay (magnification: 200 ×). Data for **C**, **D**, **F** is presented as mean ± SEM, n = 6, ***P < 0.001, **P < 0.01, *P < 0.05

The positive outcomes of the above experiments implied that PTX@GCA-NPs were expected to prolong the residue time in the GIT and enhance permeability across the intestine via the ASBT-mediated pathway. Subsequently, we performed a pharmacokinetic study to confirm this phenomenon. The PTX concentration in the PTX@GCA-NPs group was higher than that in the other orally administered groups (Fig. 7A, B). Additional file 1: Table S3 displays the detailed pharmacokinetic parameters. The AUC_0-∞_ of the PTX@GCA-NPs group was 19- and 4-fold higher than that of the Taxol^®^ (oral group, p.o.) and PTX@NPs groups, respectively. The peak concentration (C_max_) of the PTX@GCA-NPs group was 12- and 4-fold higher than that of the Taxol^®^ (p.o.) and PTX@NPs groups, demonstrating that GCA-coated nanoparticles could dramatically improve the oral bioavailability of PTX. To investigate the role of GCA in mediating intestinal transport, 50 mg/kg TCA was injected subcutaneously to competitively inhibit ASBT activity. As shown in Additional file 1: Table S3, a threefold decrease in AUC_0-∞_ was observed after TCA pretreatment compared with that of rats without TCA pretreatment, revealing the importance of ASBT in increasing the bioavailability of PTX. According to previous research, after endocytosis by intestinal epithelial cells, bile acid-decorated nanoparticles are transported into the lymphatic system and then absorbed into the systemic circulation, avoiding the first-pass effect in the liver [18, 44]. This characteristic of the bile acid-ASBT pathway is important for enhancing oral absorption of drugs. Therefore, we verified whether the prepared GCA-NPs could be transported into the lymphatic circulation by subcutaneously injecting rats with cycloheximide (CHE) at a dose of 3.6 mg/kg before oral administration of PTX@GCA-NPs. Compared with the rats that did not receive CHE, the AUC_0-∞_ and C_max_ showed a 9- and 11-fold decline, respectively, confirming the role of lymphatic circulation in drug absorption. The results of the in vivo pharmacokinetic study correlated with those of the in vitro experiments. The dramatic enhancement of oral bioavailability demonstrated that GCA-NPs were able to prolong the retention time and improve the penetration rate of PTX in the intestine, owing to their mucoadhesive properties, ASBT-targeting, and P-gp and lysosome inhibition.

### In vivo anti-cancer efficacy study of PTX@GCA-NPs

LL2 lung cancer-bearing mice model was established to evaluate the tumor inhibition efficacy and overall survival after oral administration of PTX@GCA-NPs. In the tumor inhibition study, xenografted lung cancer-bearing mice were divided into seven groups and treated with various preparations. The tumor volume curve showed rapid tumor growth after treatment with 5% glucose, while the Taxol^®^ (10 mg/kg, p.o.) group showed insignificant antitumor efficacy (Fig. 7C and Additional file 1: Fig. S7). Furthermore, when PTX was encapsulated into NPs without bile acid decoration (PTX@NPs) (10 mg/kg, p.o.), the tumor inhibition effect was improved, but was less than that of nanoparticles with bile acid conjugation (PTX@GCA-NPs). Encouragingly, obvious antitumor effects were observed after oral administration of PTX@GLNPs (10 mg/kg, p.o.), Taxol^®^ (10 mg/kg, i.v.), and different doses of PTX@GCA-NPs (low dose: 5 mg/kg, high dose: 10 mg/kg, p.o.), demonstrating that bile acid decoration on the surface of nanoparticles is crucial for enhancing the antitumor efficacy of orally administered PTX. In addition, PTX@GLNPs exhibited lower tumor inhibitory activity than PTX@GCA-NPs. Therefore, we speculated that the introduction of Qu into the delivery system could improve the antitumor efficiency by inhibiting the biological functions of P-gp.

Notably, at a low dose (5 mg/kg, p.o.), the PTX@GCA-NP group showed antitumor efficacy similar to that of Taxol^®^ (10 mg/kg, i.v.). A better antitumor effect after intragastric administration of a high dose of PTX@GCA-NPs (10 mg/kg) was observed, compared to the intravenous group. To further evaluate the chemotherapeutic efficacy of PTX@GCA-NPs, tumors were excised and weighed 2 days after the final oral treatment (day 19 post-inoculation). As shown in Fig. 7D, E, the average tumor weights of mice receiving different treatments were in the order of 5% glucose > Taxol^®^ (10 mg/kg, p.o.) > PTX@NPs (10 mg/kg, p.o.) > PTX@GLNPs (10 mg/kg, p.o.) > PTX@GCA-NPs (5 mg/kg, p.o.) > Taxol^®^ (10 mg/kg, i.v.) > PTX@GCA-NPs (10 mg/kg, p.o.), consistent with the antitumor curve.

The TUNEL assay was conducted to study chemotherapy-induced apoptosis in tumor tissues. As shown in Fig. 7F and Additional file 1: Fig. S8, a high apoptosis rate was observed in the orally administered PTX@GCA-NP (10 mg/kg) group, consistent with the results of the tumor inhibition study. Therefore, we concluded that orally administered with PTX@GCA-NPs at a dose of 10 mg/kg once every two days could suppress tumor development and exhibited better chemotherapeutic efficacy than intravenous Taxol^®^. These exciting results were perhaps due to immune activation in the tumor environment induced by metronomic chemotherapy, which is a low-dose chemotherapy regimen with shorter dosing intervals expected to elicit a better antitumor effect owing to its potential to regulate the tumor environment and activate immune responses [45–47].

### In vivo safety evaluation of PTX@GCA-NPs

**Fig. 8:**
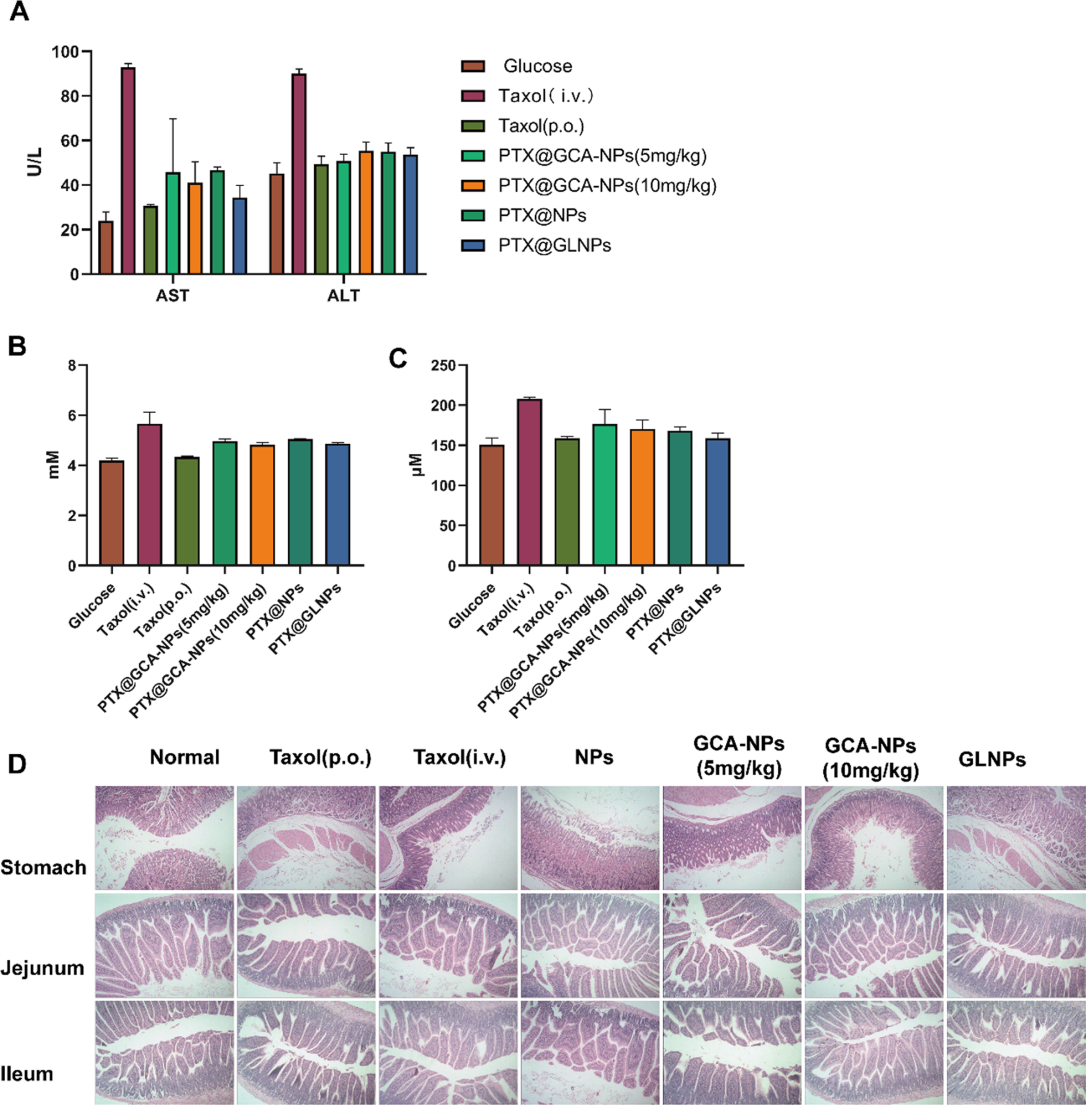
In vivo safety evaluation of oral PTX@GCA-NPs. Enzyme activities of **A** Aspartate transaminase (AST), Alkaline aminotransferase (ALT), **B** Blood urea nitrogen (BUN) and **C** Creatine (CRE) in plasma serum. Data is presented as mean ± SEM, n = 3. **D** Histological images of stomach, jejunum and ileum dyed with H&E staining. (magnification: 200 ×)

For clinical applications, it is important to evaluate safety in vivo. The body weights of the mice were measured every two days. The body weight in the Taxol^®^ (i.v.) group exhibited a slight decrease after 12 days of treatment, whereas no obvious change was observed in the other groups (Additional file 1: Fig. S9A), implying that no significant systemic toxicity was caused by orally administered nanoparticles. Furthermore, nephrotoxicity has been reported in patients treated with Taxol^®^ because of the presence of Cremophor EL (CrEL), which is a risk factor associated with adverse effects [48]. Therefore, we analyzed hepatotoxicity and nephrotoxicity by assaying enzyme activity in the serum after the administration of various preparations. After 6-time oral administration and 3-time intravenous injection, the blood of mice was collected on day 12 post-treatment (Fig. 8A–C). Compared with the 5% glucose group, there was no significant increase in the corresponding markers of enzyme activity (hepatotoxicity: aspartate transaminase (AST) and alanine transaminase (ALT); nephrotoxicity: blood urea nitrogen (BUN) and creatinine (CRE)) among orally treated groups, whereas enzyme levels were markedly increased in the Taxol^®^ (i.v.) group, confirming that the prepared PTX@GCA-NPs alleviated the in vivo toxicity of Taxol^®^.

H&E staining of tissues and organs was performed to characterize the safety of nanoparticles in major tissues and organs (Fig. 8D, Additional file 1: Fig. S9B). Two days after the final oral administration, all mice were euthanized and their organs were collected. Considering the importance of gastrointestinal safety for oral drugs, we collected stomach and ileum segments of the mice. The histological structures of the stomach and ileum segments separated from mice that received chemotherapy were similar to those of untreated mice, indicating that PTX@GCA-NPs are a safe formulation for the oral administration of PTX.

## Conclusion

In this study, we constructed novel ASBT-mediated endocytosis pathway-simulated biomimetic oral nanocarriers. In our study, GCA-NPs were expected to remarkably enhance the oral bioavailability of drugs owing to the enhanced intestinal epithelial cell endocytosis and permeability via the ASBT-mediated pathway after GCA decoration, Qu-mediated effective P-gp inhibition to increase the accumulation of PTX in the intestinal epithelial cells, and prolonged retention effect owing to the presence of the mucoadhesive material COS. Based on our experiments, the PTX@GCA-NPs maintained a stable particle size when passed through the GIT. PTX@GCA-NPs were internalized into epithelial cells via the ASBT-mediated pathway. Moreover, GCA-NPs were able to escape from endosomes and reduced the efficiency of the P-gp efflux pump. After being transported into the systemic circulation, the AUC_0-∞_ of PTX@GCA-NPs showed a 19-fold increase compared with that of oral Taxol^®^. Furthermore, PTX@GCA-NPs showed superior antitumor efficacy compared with intravenous injection of Taxol^®^ at an appropriate dosing regimen. We believe that these novel ASBT-mediated nanoparticles have potential application in oral delivery of hydrophobic anticancer drugs and have the potential to be used in the treatment of a variety of diseases.

## Materials and methods

### Materials

PTX, glycocholic acid (GCA), monensin, brefeldin A, chlorpromazine, amiloride, M-β-CD, and chloroquine were purchased from Dalian Meilun Biotechnology Co., Ltd (Liaoning, China). 1,2-Distearoyl-sn-glycero-3-phosphoethalamine-N-[methoxy(polyethyleneglycol)-2000](DSPE-PEG_2000_-OMe), 2-distearoyl-sn-glycero-3-phosphoethalamine-N-[(polyethyleneglycol)-2000]-carboxylic acid (DSPE-PEG_2000_-COOH), and SPC were purchased from AVT Shanghai Pharmaceutical Tech Co., Ltd (Shanghai, China). Cholesterol, quercetin, verapamil, TCA, and Cou-6 were purchased from J&K Scientific Co. Ltd. Lyso tracker, endoplasmic reticulum (ER) tracker, Golgi complex tracker, and DiR were purchased from Beyotime Institute of Biotechnology Co., Ltd (Jiangsu, China). ASBT rabbit polyclonal antibody (anti-ASBT) and Alexa 594^®^ goat anti-rabbit secondary antibodies were purchased from Abcam PLC (Cambridge, MA, USA). DAPI, 4% paraformaldehyde, Triton X-100, BCA kit, and RIPA lysis buffer were purchased from Solarbio Life Sciences Co. Ltd (Beijing, China). Pepsin and trypsin were purchased from Sigma-Aldrich (St. Louis, MO, USA).

### Synthesis of GCOS and DQ

GCOS was synthesized by a conjugation reaction between the carboxyl groups of chitosan oligosaccharide (COS) and the amino groups of GCA. The process for the synthesis of GCOS is shown in Additional file 1: Fig. S1. Briefly, GCA (60 mg), N-hydroxysuccinimide (NHS) (40 mg), and 1-(3-dimethylaminopropyl)-3-ethylcarbodiimide hydrochloride (EDC) (150 mg) were dissolved in 28 mL of DMSO, and COS (160 mg) was dissolved in 4 mL of deionized water and diluted with 8 mL of DMSO to form a COS/DMSO mixed solution. The COS/water/DMSO mixed solution was dropped into the GCA solution under stirring. After stirring at 25 °C for 24 h, the resulting solution precipitated in acetone. The precipitates were collected by centrifugation, washed twice with acetone and ethanol, and dried under vacuum. The presence of GCOS was confirmed using ^1^H NMR, FT-IR spectroscopy and MALDI-TOF.

Quercetin-modified DSPE-PEG_2000_ was synthesized from DSPE-PEG_2000_-COOH (100 mg), quercetin (14 mg), EDC (130 mg), and NHS (80 mg) dissolved in 25 mL of DMSO and then stirred for 24 h at 25 °C. At the end of the reaction, the resulting solution was transferred into a dialysis bag (MWCO 2.5 kDa) and dialyzed against deionized water for 72 h to remove unreacted substances and reaction solvent. Subsequently, the resulting solution was vacuum-dried. The structure of DQ was confirmed using ^1^H NMR, FT-IR and MALDI-TOF.

### Synthesis of PTX-cholesterol complex

PTX: cholesterol in a molar ratio of 1:1 was dissolved in acetone and stirred at 40 °C for 2 h under nitrogen [47]. The resulting solution was rotated at 40 °C for 30 min to obtain dried PTX-CHOL. Subsequently, PTX, cholesterol, a physical mixture of PTX and cholesterol, and PTX-CHOL were characterized using DSC analysis (TA Instruments Inc., Sherman, TX, USA).

### Preparation of PTX-loaded nanoparticles

PTX-loaded quercetin-modified liposomes (PTX@QL) were prepared using a thin-film hydration method. SPC (250 mg), cholesterol (10 mg), DSPE-mPEG_2000_ (38 mg), DQ (12 mg), and PTX-CHOL (8.7 mg) were dissolved in chloroform. The solvent was removed using a rotary evaporator and vacuum drying oven to form uniform lipid films [49, 50], which were then hydrated by 5% glucose injection. The hydrated fluids were sonicated in an ice bath for 10 min (65 W) to obtain small-sized quercetin-modified PTX-loaded liposomes.

PTX-loaded GCOS-modified nanoparticles (PTX@GCA-NPs) were prepared using the electrostatic binding method. Briefly, a GCOS stock solution (24 mg/mL) was prepared by dissolving GCOS in a 5% glucose injection. The solutions were passed through an 8 μm filter to remove impurities. The GCOS stock solution was dropped into the PTX@QL solution and stirred for 20 min at room temperature, followed by 30 min of incubation without stirring. PTX-loaded GCA-NPs were stored at 4 °C. COS was used instead of GCOS to prepare PTX-loaded nanoparticles without GCA decoration (PTX@NPs) using the above method. PTX-loaded GCA-modified nanoparticles without Qu (PTX@GLNPs) were prepared without DQ using the same method.

### Characterization of PTX@GCA-NPs

The particle size, polydispersity index (PDI), and zeta potential were measured using a Zetasizer Nano ZS90 instrument (Malvern Instruments Ltd., Malvern, UK). The morphology of PTX-loaded GCA-NPs was observed using a transmission electron microscope (Hitachi H-7650, Hitachi Ltd., Tokyo, Japan).

The entrapment efficiency was measured using HPLC (Agilent 1260 Infinity; Agilent Technologies, Santa Clara, CA, USA). Briefly, 500 μL of PTX-loaded GCA-NPs was mixed with 2 mL of methanol to disrupt the structure of nanoparticles entirely; after vortexing and sonicating, the solution was centrifuged to obtain the supernatant, which was injected into an HPLC C18 column (4.6 × 250, 5 μm; Agilent); the mobile phase was methanol:acetonitrile:water (23:36:41) at a flow rate of 1 mL/min under 35 °C column temperature, the injection volume was 20 μL, and the detection wavelength was 227 nm. Drug encapsulation efficiency (EE%) and drug loading capacity (DL%) were calculated as follows:

$$EE\%=\frac{Weight\;of\;encapsulated\;drug}{Weight\;of\;initial\;drug}\times 100\%$$

$$DL\%= \frac{Weight\;of\;encapsulated\;drug}{Weight\;of\;nanoparticles}\times 100\%$$

### Stability of PTX@GCA-NPs in simulated gastric and intestinal fluid

The stability of PTX@GCA-NPs in SGF (0.32% pepsin at pH 1.2 HCl) and SIF (1% trypsin in pH 6.8 PBS) was analyzed using a Zetasizer Nano ZS90 instrument [51]. The particle sizes of the initial PTX@GCA-NPs were measured. Subsequently, the PTX-loaded GCA-NPs were incubated in SGF and SIF for 2 and 6 h, respectively. The particle sizes of the sample solutions were measured and recorded at the desired time points.

### In vitro PTX release

The in vitro drug release behavior of PTX@GCA-NPs was determined using the dialysis diffusion technique under different pH conditions with gentle stirring at 37 °C. To satisfy the sink condition, 0.5% Tween-80 was used as the solubilizing agent. The release medium comprised 25 mL of SGF fluid (0.32% pepsin, pH 1.2) and SIF fluid (1% trypsin, pH 6.8) [32]. PTX@GCA-NP suspension and Taxol^®^ injection solution (1 mL each) were separately placed in a dialysis bag (MWCO 12 kDa) in SGF medium for the first 2 h, and subsequently transferred into SIF medium for the following 70 h. At the desired time points, the sample (0.5 mL) was removed and replaced with the same volume of fresh media. PTX content was evaluated by HPLC after centrifugation at 12,000 rpm for 10 min.

### Cell viability study

Caco-2 cells were chosen as an in vitro model to simulate the transport and uptake of PTX@GCA-NPs in the small intestine. Therefore, a CCK-8 assay was performed to evaluate the safety of blank GCA-NPs and PTX@GCA-NPs in Caco-2 cells. Briefly, Caco-2 cells were cultured in 96-well plates at a density of 1 × 10^4^ cells/well and incubated at 37 °C with 5% CO_2_ for 24 h. Subsequently, Caco-2 cells were treated and cultured in different concentrations of blank GCA-NPs for 24 h and PTX@GCA-NPs for 4 h. At predetermined time points, the culture medium containing GCA-NPs was replaced with the CCK-8 reagent. After incubation for another 2 h, absorbance was measured at 490 and 650 nm using a Synergy H1 Microplate Reader (BioTek, Dallas, TX, USA). Untreated cells were used as the control group, and CCK-8 wells served as the adjusted groups. Cell viability was calculated as follows:

$$Cell\;viability\times 100\%= \frac{({OD}_{sample}-{OD}_{adjust})}{({OD}_{control}-{OD}_{adjust})}\times 100\%$$

### Cellular uptake studies

Caco-2 monolayers in the Transwell^®^ membrane with a TEER over 800 Ω/cm^2^ were chosen for this experiment. First, Caco-2 cells at the density of 1 × 10^5^ cells/well were cultured on the polycarbonate membrane filter (pore size: 0.4 μm; growth area: 1.12 cm^2^) of Transwell^®^ 12-well plates for 21 days [32]. The culture medium was replaced every 1–2 days until the TEER value exceeded 800 Ω/cm^2^. Before the experiments, Caco-2 monolayers were washed thrice with pre-warmed PBS (pH 7.4).

Coumarin-6 loaded GCA-NPs (Cou-6@GCA-NPs) were prepared to examine the cellular uptake of GCA-NPs in Caco-2 monolayers. Cou-6@GCA-NPs and Cou-6@NPs were diluted with cell culture medium, DMEM without fetal bovine serum, to 10 μg/mL and added to the apical side (AP), whereas the basolateral side (BL) was replaced with fresh DMEM without fetal bovine serum. After 1 h of incubation, the Caco-2 monolayers were washed twice with cold PBS. For the quantitative study, Caco-2 monolayers were disrupted using 150 μL RIPA lysis buffer for 30 min in an ice bath. The cell lysis solution was then collected and centrifuged to obtain the supernatant. The amount of Cou-6 in Caco-2 monolayers was detected using the Synergy H1 Microplate Reader, and the amount of total protein in Caco-2 cells was determined using the BCA kit. The cellular uptake efficiency was evaluated as follows:

$$Cellular\;uptake\;efficiency \;(ug/mg)= \frac{Amount\;of\;Cou-6\; (ug)}{Amount\;of\;total\;protein \;(mg)}$$

CLSM was carried to visualize the uptake of GCA-NPs in Caco-2 monolayers. ASBT-mediated endocytosis of GCA-NPs was investigated using immunofluorescence. Briefly, cell monolayers were incubated with 10 μg/mL Cou-6@GCA-NPs and Cou-6@NPs for 1 h, washed thrice with cold PBS, and fixed with 4% paraformaldehyde for 15 min at room temperature. Cell monolayers were then stained with anti-SLC10A2 rabbit polyclonal antibody (Abcam, Cambridge, MA, USA) and detected with Alexa Fluor^®^ 647-labeled goat anti-rabbit secondary IgG (Abcam, Cambridge, MA, USA). The nuclei were stained with 5 μg/mL DAPI. Finally, the membrane was cut and observed using CLSM.

### Endocytosis pathway studies

To analyze the endocytosis pathway of GCA-NPs, 20 μg/mL chlorpromazine, 5 mg/mL M-β-CD, 25 μg/mL brefeldin, 80 μg/mL monensin, 30 μg/mL amiloride, 4.8 μg/mL chloroquine, and 100 μM TCA were incubated with Caco-2 monolayers for 1 h. Various inhibitor solutions were then replaced with Cou-6@GCA-NPs diluted in DMEM without fetal bovine serum at 10 μg/mL. After incubation for 1 h, the monolayers were washed twice with cold PBS and disrupted using RIPA lysis buffer for 30 min. The supernatant of the cell lysis solution was collected by centrifugation, and the amount of Cou-6 and total protein were detected using the Synergy H1 Microplate Reader and BCA kit, respectively.

### Trans-epithelial transport of PTX-loaded GCA-NPs

The cellular uptake efficiency of the GCA-NPs was studied in Caco-2 monolayers. Before each experiment, Caco-2 monolayers were washed twice with pre-warmed PBS (pH 7.4) and 0.5 mL each of PTX@GCA-NPs (100 μg/mL) and PTX@NPs (100 μg/mL) were added to the AP [43]. At predetermined time points (30, 60, 90, and 120 min), 300 μL of the sample was collected from the BL and supplemented with the same volume of fresh medium. The collected samples were mixed with 300 μL of methanol and centrifuged at 12,000 rpm for 10 min, and the PTX concentration in the supernatant was analyzed by HPLC. The apparent permeability coefficient (Papp) was calculated as follows:

$$Papp=dQ/(dt\times A\times {C}_{0})$$

where dQ/dt is the rate of PTX transport from the donor to the receiver chamber, A is the membrane surface area of the chamber, and C_0_ is the initial concentration of PTX.

### Trans-epithelial transport pathway studies of GCA-NPs

Cou-6@GCA-NPs were used to analyze the transport pathways of GCA-NPs in the Caco-2 monolayers. Briefly, Caco-2 monolayers were washed twice with PBS (pH 7.4), incubated with 20 μg/mL chlorpromazine, 5 mg/mL M-β-CD, 25 μg/mL brefeldin, 80 μg/mL monensin, 30 μg/mL amiloride, 4.8 μg/mL chloroquine, 10 mg/mL TCA, and 50 μg/mL verapamil for 1 h, and replaced with Cou-6@GCA-NPs that was diluted to 10 μg/mL using DMEM. The solution on the BL side was collected and the fluorescence intensity was measured using the Synergy H1 Microplate Reader.

### GCA-NPs integrity evaluation after penetrating Caco-2 monolayers

The integrity of GCA-NPs after penetration through Caco-2 cell monolayers was analyzed using FRET technology. DiO and DiI were chosen as FRET pairs and loaded into GCA-NPs (DiO/DiI@GCA-NPs) [43, 52]; the concentration of the two dyes was 100 μg/mL. The FRET signal was assayed using a fluorospectrophotometer at an excitation wavelength of 480 nm and detection range of 500–600 nm. To verify the occurrence of FRET based on integral nanoparticles, the fluorescence spectra of the DiO/DiI@GCA-NPs diluted in deionized water and acetone were measured.

Caco-2 cell monolayers were washed twice with prewarmed PBS. Subsequently, the DiO/DiI@GCA-NPs and DiO@GCA-NPs + DiI@GCA-NPs physical mixture solutions (control group) were added to the AP side. The fluorescence spectrum of the BL side was measured at regular intervals (n = 3). The FRET efficiency was calculated as follows:

$$FRET\;ratio \left(\%\right)=\frac{{I}_{R}}{\left({I}_{G}+{I}_{R}\right)}\times 100\%$$

where I_R_ and I_G_ represent the fluorescence intensities at 501 nm and 565 nm, respectively.

### Colocalization studies of GCA-NP with endoplasmic reticulum, Golgi complex, and lysosomes

Caco-2 cells were seeded into 12-well plates at a density of 1 × 10^5^ cells/well. After culturing for 24 h, the medium was replaced with 10 µg/mL coumarin-loaded nanoparticles and incubated with monolayers. The cells were washed thrice with cold PBS. Then, various tracker probes were added and incubated for 30 min under different conditions: Lysosome Tracker Red at 37 °C, ER Tracker Red at 37 °C, and Golgi Tracker Red at 4 °C [43]. Afterwards, the tracker solutions were removed and the cells were washed three times with cold PBS. Cell nuclei were stained with 10 μg/mL Hoechst 33,342. Images were obtained using CLSM.

### Animal studies

All animal experiments were performed according to the guidelines of the Institutional Animal Care and Use Committee (IACUC). Sprague–Dawley (SD) rats (male, 150–180 g), BALB/c nude mice (male, 4–6 weeks), ICR mice (male, 4–6 weeks), and C57BL/6 mice (male, 4–6 weeks) were provided by Vital River Laboratory Animal Technology Co., Ltd. (Beijing, P.R. China). To establish lung cancer xenograft models, LL2 cells were dispersed in cold PBS and subcutaneously injected into the left right axillary fossa of C57BL/6 mice.

### In vivo biodistribution and retention study

ICR mice were used to assess the in vivo biodistribution of orally administered nanoparticles. The DiR probe was encapsulated into GCA-NPs and NPs. ICR mice were fasted overnight and only water was allowed. Then, the mice were administered DiR-loaded GCA-NPs (DiR@GCA-NPs), DiR-loaded NPs (DiR@NPs), and DiR-loaded QL (DiR@QL) via oral gavage (n = 3, 2.5 mg/kg of DiR). At 2, 8, and 24 h post treatment, the mice were euthanized, their intestines were removed and washed twice with cold normal saline, and the intestinal samples were observed by ex vivo fluorescence imaging (Caliper Life Sciences, Hopkinton, MA, USA). The fluorescence intensity in the distal ileum was analyzed using Living Image software.

### ASBT-mediated in vivo enterocyte absorption in the ileum

The absorption of Cou-6@GCA-NPs and Cou-6@NPs was observed using CLSM. The BABL/c nude mice were fasted overnight with free access to water [18]. Cou-6@GCA-NPs and Cou-6@NPs (10 mg/kg of Cou-6) were administered orally. After 2 h, the mice were euthanized, and a 2 cm segment of the distal ileum was removed and washed twice with cold normal saline. The distal ileum was embedded in OCT compound and frozen in liquid nitrogen. Then, the ileum tissue was sliced into 10 μm pieces and fixed with 4% paraformaldehyde. Immunofluorescence staining was performed to label ASBT expressed on the surface of enterocytes and the nuclei of enterocytes were stained using DAPI. The ileum was visualized using CSLM.

### In vivo pharmacokinetics study

The SD rats were fasted overnight and allowed water only. Rats were randomized into six groups (n = 3). To study the role of lymphatic circulation and ASBT in GCA-NPs absorption in vivo, rats were subcutaneously injected with 3.6 mg/kg of CHE and orally administered 50 mg/kg of TCA solution [18, 39]. After 30 min, SD rats were orally administered with Taxol^®^, PTX@GCA-NPs, or PTX@NPs at a dose of 10 mg/kg or intravenously injected with Taxol^®^ at a dose of 10 mg/kg. Blood samples (0.5 mL) were collected from the retroorbital plexus at predetermined time points. Plasma was collected after centrifugation at 4000 rpm for 10 min and stored at − 80 ℃ until further analysis. Plasma PTX levels were analyzed using HPLC–MS/MS.

### In vivo anticancer efficacy

LL2 tumor-bearing mice model was established. When the tumor volume reached 100 mm^3^, the mice were randomly divided into seven groups (n = 6): 5% glucose, Taxol^®^ (p.o., 10 mg/kg), Taxol^®^ (i.v., 10 mg/kg), PTX@GCA-NPs (p.o., 10 mg/kg), PTX@GCA-NPs (p.o., 5 mg/kg), PTX@NPs (p.o., 10 mg/kg), and PTX@GLNPs (p.o., 10 mg/kg). The mice were orally administered various preparations twice daily, except the intravenous group which was administered preparations every four days. Body weight and tumor volume were recorded every two days. On day 12, the mice were euthanized and the tumors and main organs were surgically removed. Apoptosis of tumor cells was analyzed using the TUNEL assay.

### In vivo toxicity study

LL2 lung cancer-bearing mice were administered 5% glucose, Taxol^®^ (p.o., 10 mg/kg), Taxol^®^ (i.v., 10 mg/kg), PTX@GCA-NPs (p.o., 10 mg/kg), PTX@NPs (p.o., 10 mg/kg), or PTX@GLNPs (p.o., 10 mg/kg). The mice were orally administered various preparations twice daily, except the intravenous group which was administered preparations every four days. After final administration, 0.3 mL of blood sample was collected (n = 3). Serum ALT and AST levels were analyzed to determine in vivo hepatotoxicity, and serum BUN and CRE levels were measured to evaluate nephrotoxicity [32]. The major organs were stained with hematoxylin and eosin (H&E).

### Statistical analysis

All data are presented as the mean ± standard error of mean (SEM). Student’s t-test was used to evaluate statistical differences between two groups. Statistical analysis was performed using GraphPad prism software, and a P value < 0.05 was regarded as statistically significant.

## Supplementary Information

**Additional file 1: Fig. S1**. Synthetic route of A DSPE-PEG2000-modified quercetin (DQ) and B GCA-modified COS (GCOS). **Fig. S2**. MALDI-TOF spectrum of chitosan oligosaccharide (COS) and GCOS. **Fig. S3**. DSC scanning results of A cholesterol. B PTX. C PTX-CHOL complex. **Fig. S4**. Cell cytotoxic evaluation of PTX@GCA-NPs. Cell viability of Caco-2 cells after incubated with A PTX@GCA-NPs for 4 h and B Blank GCA-NPs for 24 h. Data is presented as mean ± SEM, n = 6. **Fig. S5**. Changes of TEER value compared after Caco-2 cell monolayers were incubated with PTX@GCA-NPs and PTX@NPs, respectively. Data is presented as mean ± SEM, n =3. **Fig. S6**. FRET phenomenon validation. FRET signal of A DiO/DiR@GCA-NPs dispersed with deionized water and acetone. B FRET efficiency of DiO/DiI@GCA-NPs mixed with acetone and deionized water. C DiO/DiI@GCA-NPs, DiO@GCA-NPs, DiI@GCA-NPs. (Excitation wavelength at 480 nm and detection at 500 – 600 nm). **Fig. S7**. Relative tumor volume of LL2 bearing mice. Date is presented as mean ± SEM, n = 6. **Fig. S8**. Apoptosis rate analysis of TUNEL assay. Data is presented as mean ± SEM, n = 3, ***P < 0.001, **P < 0.01, *P < 0.05. **Fig. S9**. In vivo safety evaluation of LL2 bearing mice after dosing for 12 days. A Body weights. B Histological images of heart, liver, spleen, lung and kidney dyed with H&E staining. Date is presented as mean ± SEM, n = 6. (magnification: 200 ×). **Table S1**. Physicochemical properties of nanoparticles. Data is presented as mean ± SEM, n =3. **Table S2**. FRET efficiency of DiO/DiI@GCA-NPs and DiO@GCA-NPs + DiI@GCA-NPs mixture in BP side after incubated with Caco-2 cell monolayers. Data is presented as mean ± SD, n = 3. **Table S3**. Pharmacokinetic parameters following intravenous injection of Taxol and oral gavage of Taxol, PTX@NPs, PTX@GCA-NPs, PTX@GCA-NPs + TCA and PTX@GCA-NPs + CHE refer to the rats was orally administered with TCA and subcutaneous injected with CHE before oral PTX@GCA-NPs, respectively. (PTX: 10 mg/kg, TCA: 50 mg/kg, CHE: 3.6 mg/kg). Data is presented as mean ± SEM, n = 3.

## Data Availability

All data generated or analyzed during this study are included in this published article and its supplement files. The data that support the findings of this study are available from the corresponding author upon reasonable request.

## Abbreviations

ALT: Alanine transaminase
AP: Apical sides
ASBT: Apical sodium-dependent bile acid receptor
AST: Aspartate transaminase
BL: Basolateral sides
BUN: Blood urea nitrogen
CHE: Cyclohexanoimide
CLSM: Confocal Laser Spectrum Microscope
COS: Chitosan oligosaccharide
CPZ: Chlorpromazine
CRE: Creatinine
DSPE-mPEG_2000_: 1,2-Distearoyl-sn-glycero-3-phosphoethalamine-N-[methoxy(polyethyleneglycol)-2000]
DSPE-PEG_2000_-COOH: 1,2-Distearoyl-sn-glycero-3-phosphoethalamine-N-[(polyethyleneglycol)-2000]-carboxylic acid
EDC: 1-(3-Dimethylaminopropyl)-3-ethylcarbodiimide hydrochloride
ER: Endoplasmic reticulum
FcRn: Neonatal Fc receptor
FRET: Förster resonance energy transfer
GA: Golgi apparatus complex
GCA: Glycocholic acid
GIT: Gastrointestinal tract
IABAP: Ideal bile acid-binding protein
MDR: Multi-drug resistance
MTC: Maximum tolerable concentration
M-β-CD: Methyl-β-cyclodextrin
NHS: N-hydroxysuccinimide
NSCLC: Non-small cell lung cancer
PDI: Polydispersity index
PTX: Paclitaxel
Qu: Quercetin
SGF: Simulated gastric fluid
SGLT: Sodium-dependent glucose receptor
SIF: Simulated intestinal fluid
SPC: Soybean phospholipid
TEER: Trans-epithelial electrical resistance
