# Correction: Biomimetic and temporal-controlled nanocarriers with ileum transporter targeting for achieving oral administration of chemotherapeutic drugs

**DOI:** 10.1186/s12951-022-01744-8

**Published:** 2022-12-27

**Authors:** Wei Liu, Ying Han, Xin Xin, Liqing Chen, Yanhong Liu, Chao Liu, Xintong Zhang, Mingji Jin, Jingzhe Jin, Zhonggao Gao, Wei Huang

**Affiliations:** 1 State Key Laboratory of Bioactive Substance and Function of Natural Medicines, Institute of Materia Medica, Chinese Academy of Medical Sciences and Peking Union Medical College, Beijing, 100050 People’s Republic of China; 2 Beijing Key Laboratory of Drug Delivery Technology and Novel Formulations, Department of Pharmaceutics, Institute of Materia Medica, Chinese Academy of Medical Sciences and Peking Union Medical College, Beijing, 100050 People’s Republic of China; 3 Department of Oncology, The First Hospital of Dandong City, Dandong, 118000 Liaoning People’s Republic of China

**Correction: Journal of Nanobiotechnology (2022) 20:281** 10.1186/s12951-022-01460-3

Following publication of the original article [1], the authors identified an error in Fig. 8d. The correct Fig. 8 is given in this correction.

**Fig. 8:**
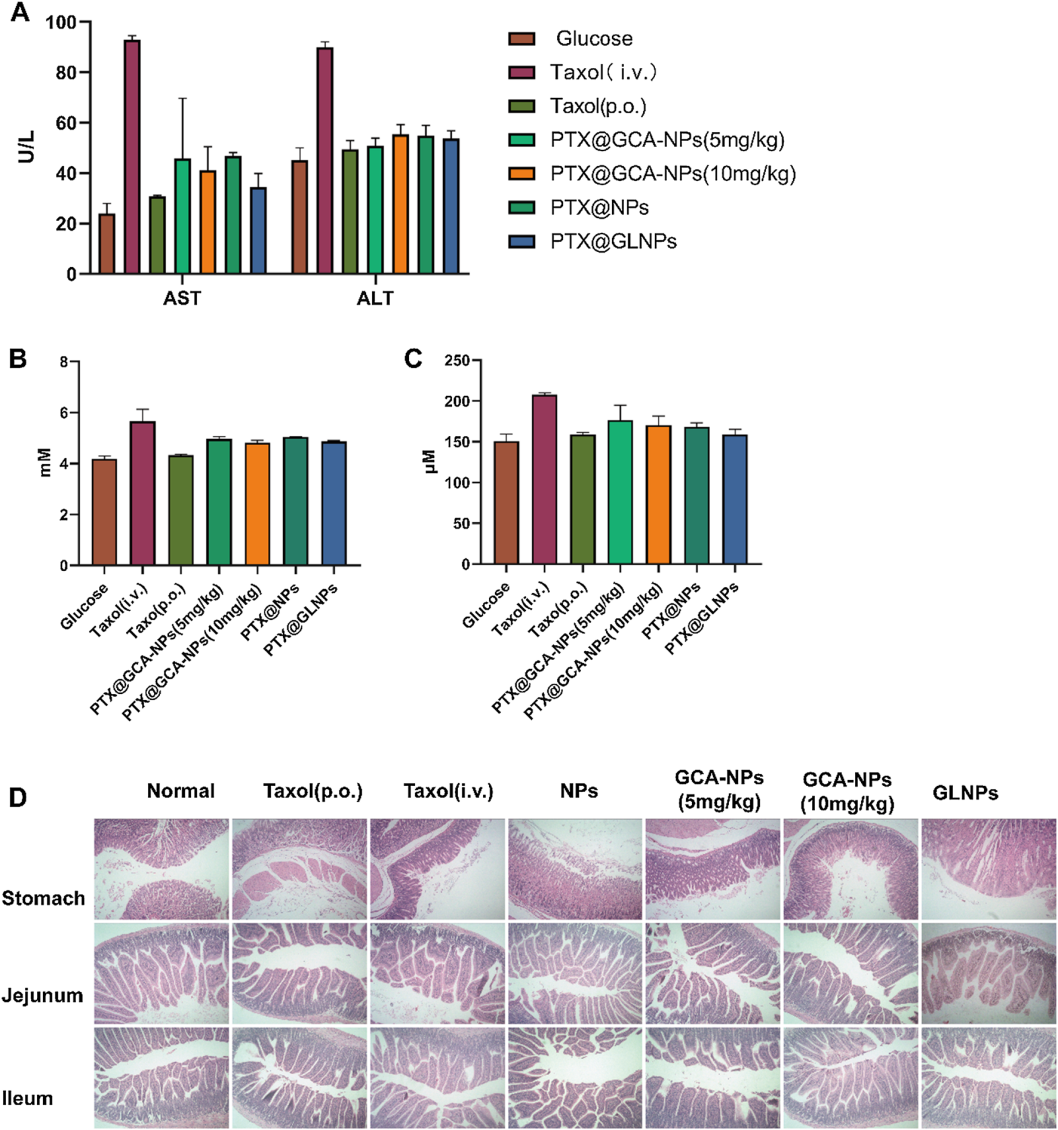
In vivo safety evaluation of oral PTX@GCA-NPs. Enzyme activities of **A** Aspartate transaminase (AST), Alkaline aminotransferase (ALT), **B** Blood urea nitrogen (BUN) and **C** Creatine (CRE) in plasma serum. Data is presented as mean ± SEM, n = 3. **D** Histological images of stomach, jejunum and ileum sectioned from mice and dyed with H&E staining. (magnification: 200 ×)

The original article [1] has been corrected.
