# Supplementary material for: Biomimetic and temporal-controlled nanocarriers with ileum transporter targeting for achieving oral administration of chemotherapeutic drugs
Source: J Nanobiotechnology. 2022 Jun 15;20:281. doi: 10.1186/s12951-022-01460-3 (PMC9199201; doi:10.1186/s12951-022-01460-3)
Supplement: Supplementary file 1 — Additional file 1: Fig. S1. Synthetic route of A DSPE-PEG2000-modified quercetin (DQ) and B GCA-modified COS (GCOS). Fig. S2. MALDI-TOF spectrum of chitosan oligosaccharide (COS) and GCOS. Fig. S3. DSC scanning results of A cholesterol. B PTX. C PTX-CHOL complex. Fig. S4. Cell cytotoxic evaluation of PTX@GCA-NPs. Cell viability of Caco-2 cells after incubated with A PTX@GCA-NPs for 4 h and B Blank GCA-NPs for 24 h. Data is presented as mean ± SEM, n = 6. Fig. S5. Changes of TEER value compared after Caco-2 cell monolayers were incubated with PTX@GCA-NPs and PTX@NPs, respectively. Data is presented as mean ± SEM, n =3. Fig. S6. FRET phenomenon validation. FRET signal of A DiO/DiR@GCA-NPs dispersed with deionized water and acetone. B FRET efficiency of DiO/DiI@GCA-NPs mixed with acetone and deionized water. C DiO/DiI@GCA-NPs, DiO@GCA-NPs, DiI@GCA-NPs. (Excitation wavelength at 480 nm and detection at 500 – 600 nm). Fig. S7. Relative tumor volume of LL2 bearing mice. Date is presented as mean ± SEM, n = 6. Fig. S8. Apoptosis rate analysis of TUNEL assay. Data is presented as mean ± SEM, n = 3, ***P < 0.001, **P < 0.01, *P < 0.05. Fig. S9. In vivo safety evaluation of LL2 bearing mice after dosing for 12 days. A Body weights. B Histological images of heart, liver, spleen, lung and kidney dyed with H&E staining. Date is presented as mean ± SEM, n = 6. (magnification: 200 ×). Table S1. Physicochemical properties of nanoparticles. Data is presented as mean ± SEM, n =3. Table S2. FRET efficiency of DiO/DiI@GCA-NPs and DiO@GCA-NPs + DiI@GCA-NPs mixture in BP side after incubated with Caco-2 cell monolayers. Data is presented as mean ± SD, n = 3. Table S3. Pharmacokinetic parameters following intravenous injection of Taxol and oral gavage of Taxol, PTX@NPs, PTX@GCA-NPs, PTX@GCA-NPs + TCA and PTX@GCA-NPs + CHE refer to the rats was orally administered with TCA and subcutaneous injected with CHE before oral PTX@GCA-NPs, respectively. (PTX: 10 mg/kg, TCA: 50 mg/kg, CHE: 3.6 mg/k [file 12951_2022_1460_MOESM1_ESM.docx]

**Biomimetic and temporal-controlled nanocarriers with ileum transporter targeting for achieving oral administration of chemotherapeutic drugs**

Wei Liu^1,2^, Ying Han^1,2^, Xin Xin^1,2^, Liqing Chen^1,2^, Yanhong Liu^1,2^, Xintong Zhang^1,2^, Chao Liu^1,2^, Mingji Jin^1,2^, Jingzhe Jin^3^, Zhonggao Gao^1,2^*, Wei Huang^1,2^*

^1^ State Key Laboratory of Bioactive Substance and Function of Natural Medicines, Institute of Materia Medica, Chinese Academy of Medical Sciences and Peking Union Medical College, Beijing 100050, P.R. China.

^2^ Beijing Key Laboratory of Drug Delivery Technology and Novel Formulations, Department of Pharmaceutics, Institute of Materia Medica, Chinese Academy of Medical Sciences and Peking Union Medical College, Beijing 100050, P.R. China.

^3^ Department of Oncology, The First Hospital of Dandong City, Liaoning province 118000, P.R. China.

^*^Correspondence: [zggao@imm.ac.cn](mailto:zggao@imm.ac.cn) (Z. Gao), [huangwei@imm.ac.cn](mailto:huangwei@imm.ac.cn) (W. Huang).


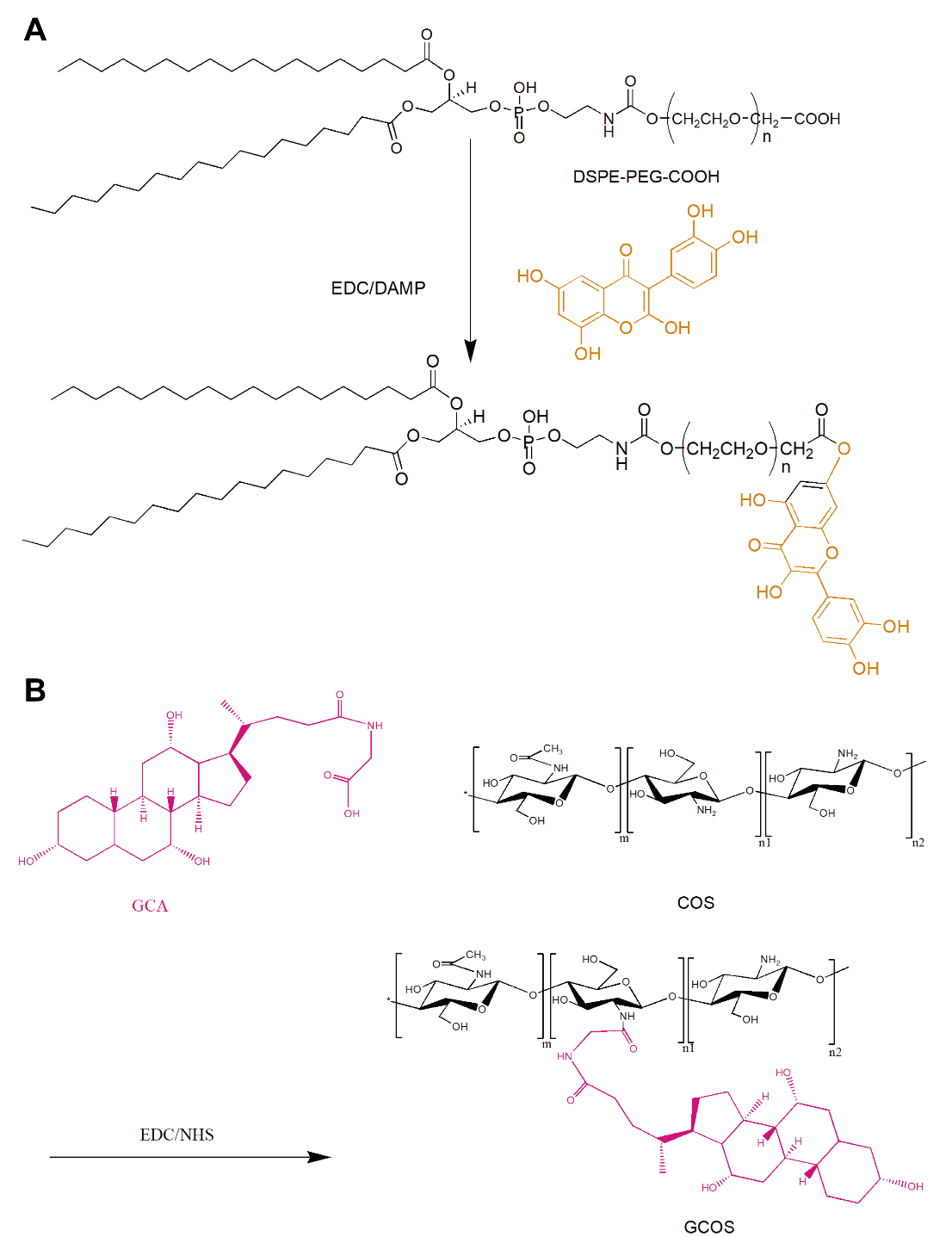


**Fig. S1**. Synthetic route of **A** DSPE-PEG_2000_-modified quercetin (DQ) and **B** GCA-modified COS (GCOS).


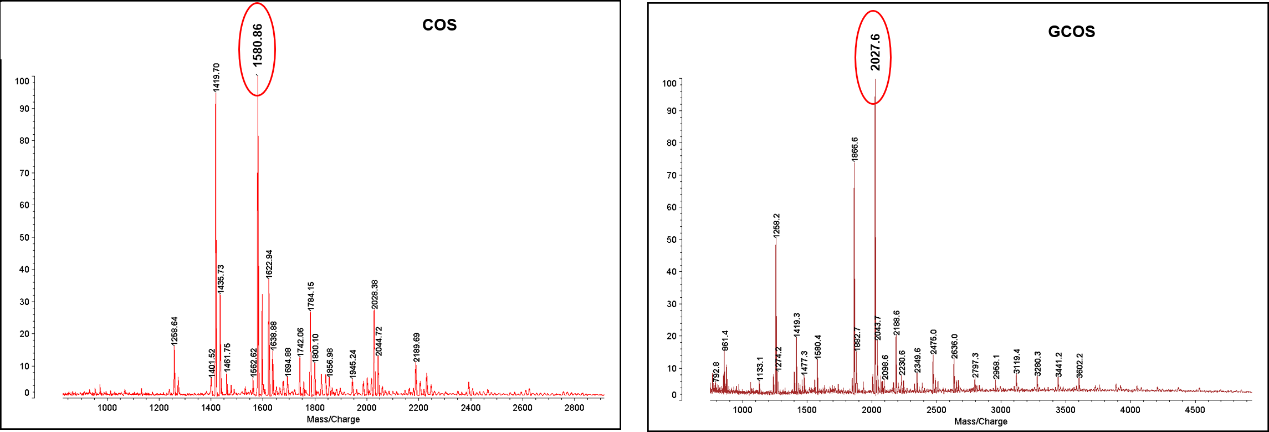


Fig. S2. MALDI-TOF spectrum of chitosan oligosaccharide (COS) and GCOS.


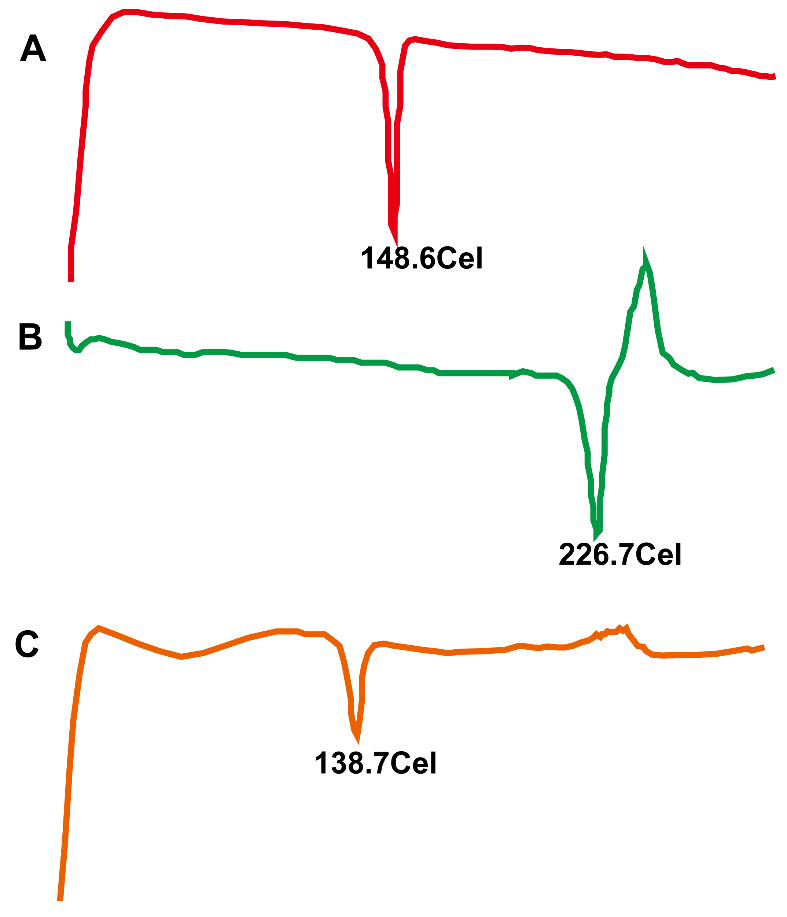


**Fig. S3.** DSC scanning results of **A** cholesterol. **B** PTX. **C** PTX-CHOL complex.


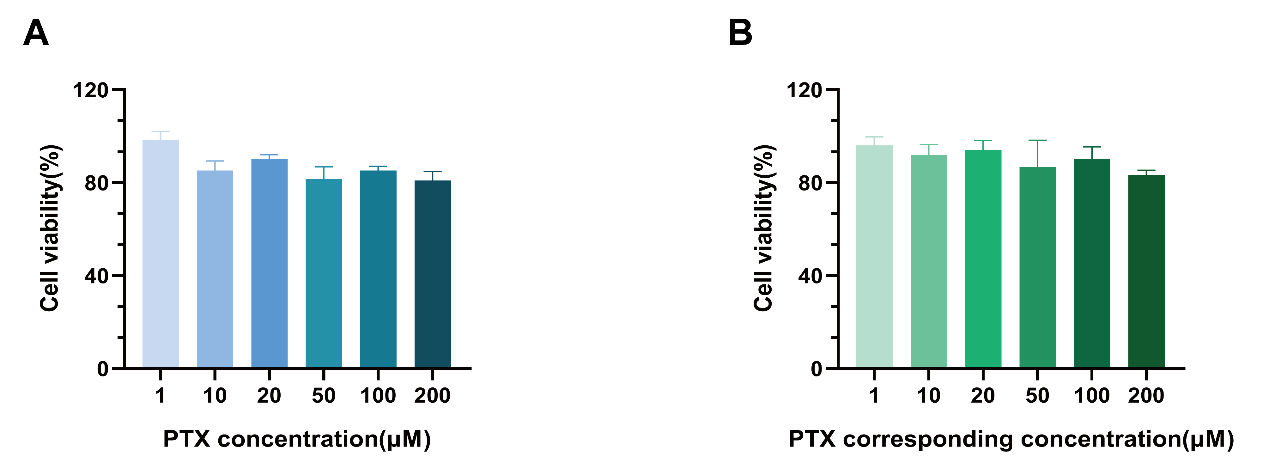


**Fig. S4**. Cell cytotoxic evaluation of PTX@GCA-NPs. Cell viability of Caco-2 cells after incubated with **A** PTX@GCA-NPs for 4 h and **B** Blank GCA-NPs for 24 h. Data is presented as mean ± SEM, n = 6.


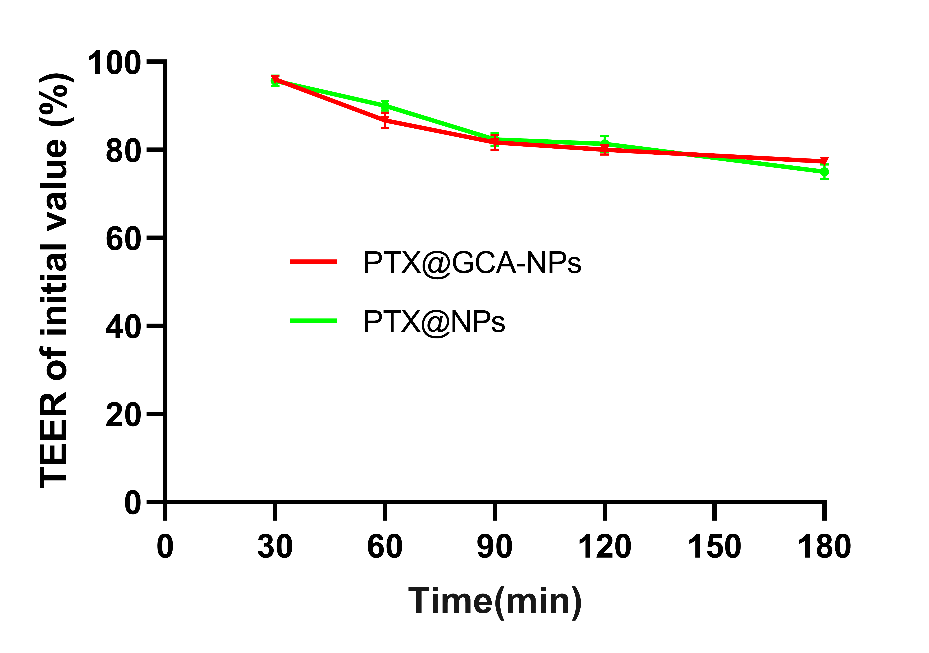


**Fig. S5.** Changes of TEER value compared after Caco-2 cell monolayers were incubated with PTX@GCA-NPs and PTX@NPs, respectively. Data is presented as mean ± SEM, n =3.


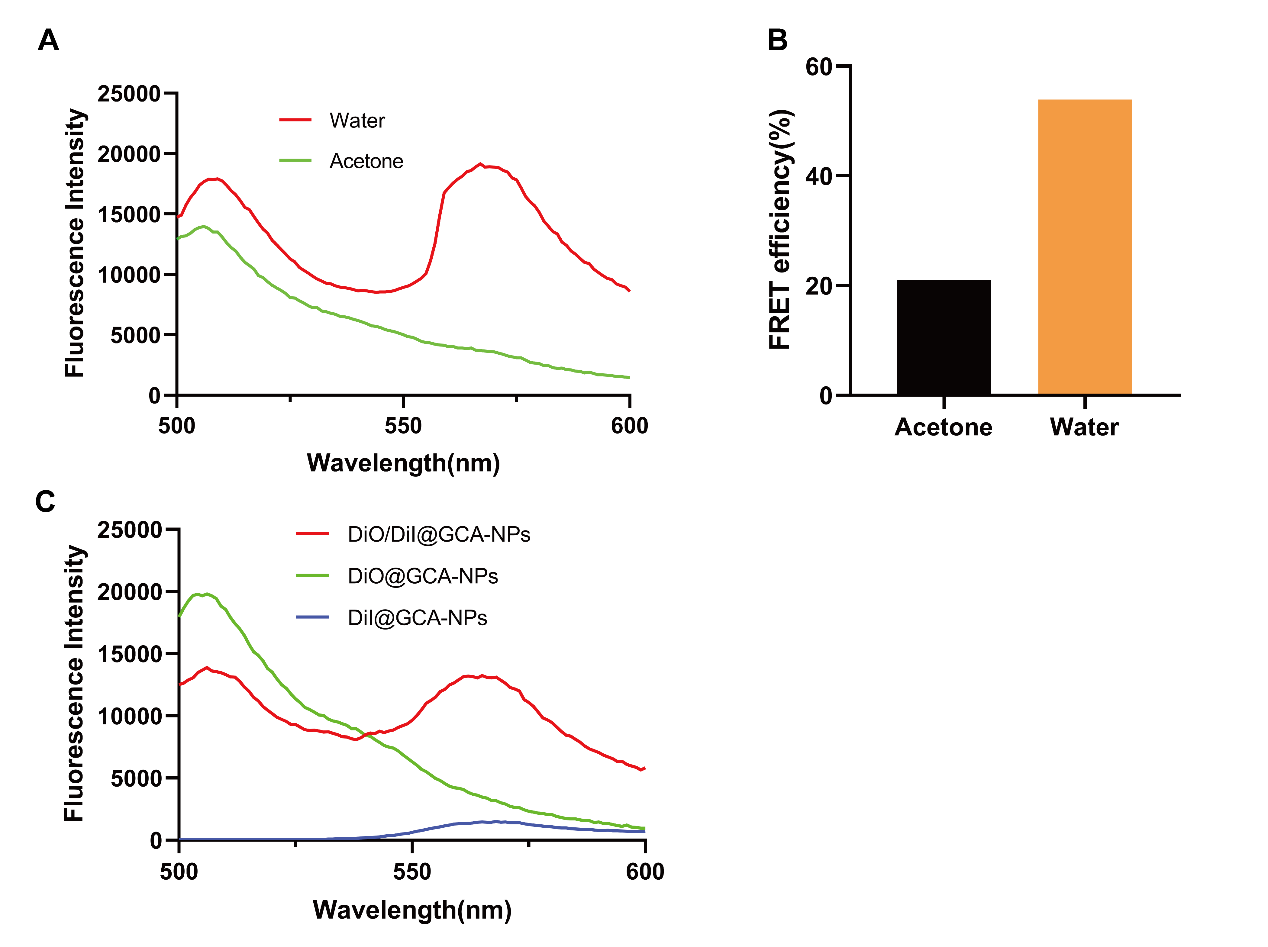


**Fig. S6.** FRET phenomenon validation. FRET signal of **A** DiO/DiR@GCA-NPs dispersed with deionized water and acetone. **B** FRET efficiency of DiO/DiI@GCA-NPs mixed with acetone and deionized water. **C** DiO/DiI@GCA-NPs, DiO@GCA-NPs, DiI@GCA-NPs. (Excitation wavelength at 480 nm and detection at 500 – 600 nm).


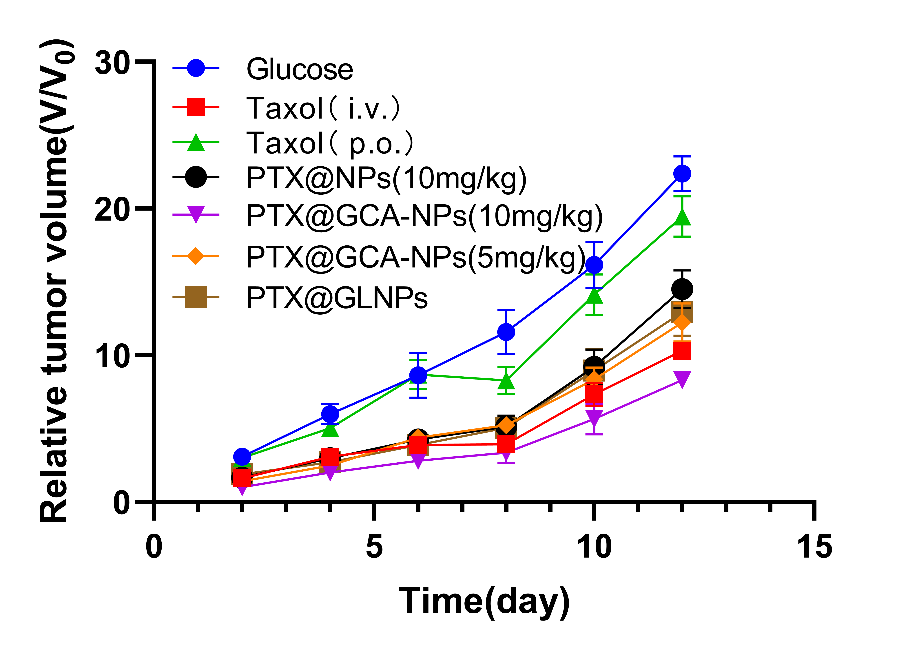


**Fig. S7.** Relative tumor volume of LL2 bearing mice. Date is presented as mean ± SEM, n = 6.


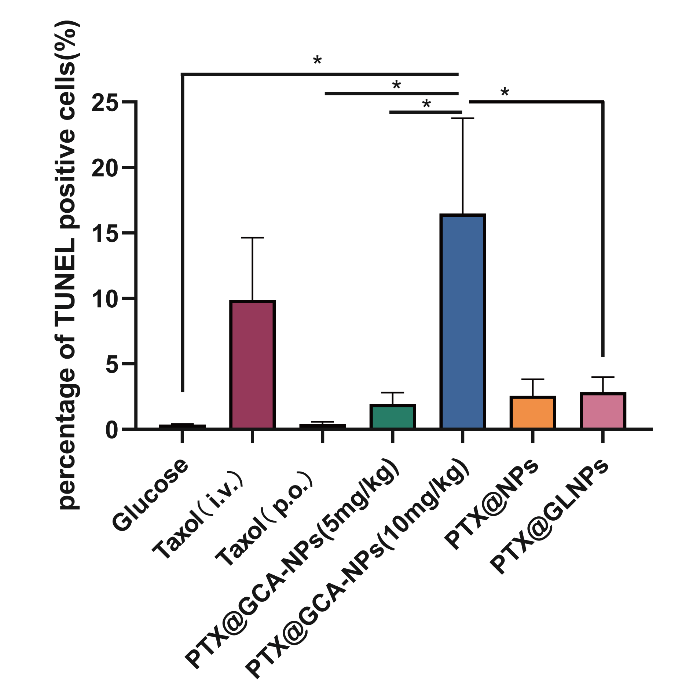


**Fig. S8.** Apoptosis rate analysis of TUNEL assay. Data is presented as mean ± SEM, n = 3, ***P < 0.001, **P < 0.01, *P < 0.05**.**


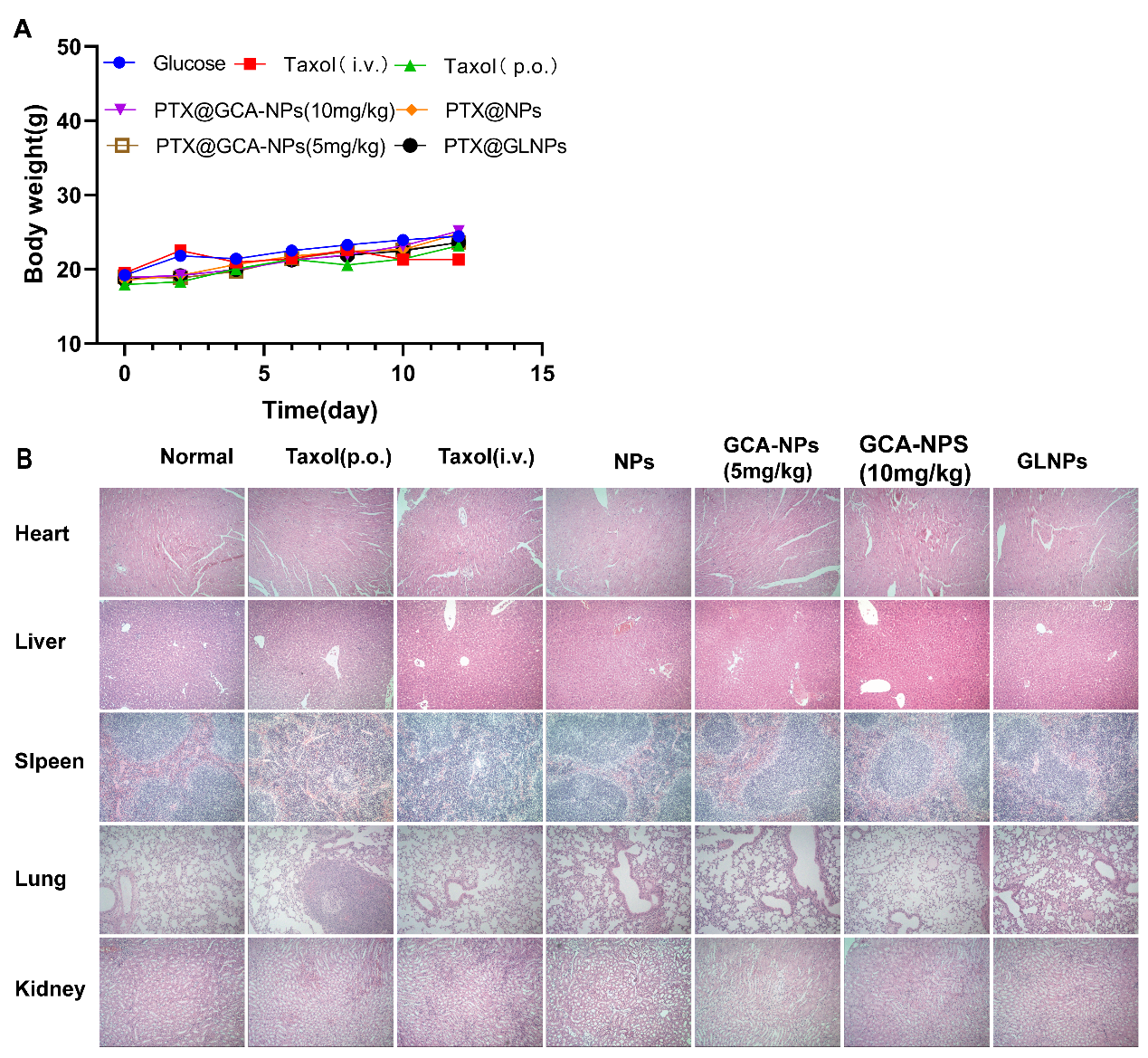


**Fig. S9.** In vivo safety evaluation of LL2 bearing mice after dosing for 12 days. **A** Body weights. **B** Histological images of heart, liver, spleen, lung and kidney dyed with H&E staining. Date is presented as mean ± SEM, n = 6. (magnification: 200 ×).

**Table S1**. Physicochemical properties of nanoparticles. Data is presented as mean ± SEM, n =3.

| Groups | Diameter (nm) | PDI | EE (%) | LC (%) |
| --- | --- | --- | --- | --- |
| PTX@GCA-NPs | 84 ± 3 | 0.22 ± 0.01 | 97.50 ± 1.69 | 1.55 ± 0.03 |
| PTX@NPs | 76 ± 2 | 0.19 ± 0.02 | 97.57 ± 0.58 | 1.55 ± 0.01 |
| PTX@LP | 118 ± 1 | 0.29 ± 0.01 | 98.58 ± 0.69 | 1.56 ± 0.01 |

**Table S2.** FRET efficiency of DiO/DiI@GCA-NPs and DiO@GCA-NPs + DiI@GCA-NPs mixture in BP side after incubated with Caco-2 cell monolayers. Data is presented as mean ± SD, n = 3.

| Time | DiO/DiI@GCA-NPs (%) | DiO@GCA-NPs + DiI@GCA-NPs (%) |
| --- | --- | --- |
| 30 min | 50.78 ± 2.09 | 29.21 ± 0.42 |
| 60 min | 50.05 ± 1.49 | 30.13 ± 0.37 |
| 120 min | 52.17 ± 1.47 | 30.31 ± 0.07 |
| 180 min | 51.97 ± 0.18 | 30.34 ± 0.35 |

**Table S3.** Pharmacokinetic parameters following intravenous injection of Taxol and oral gavage of Taxol, PTX@NPs, PTX@GCA-NPs, PTX@GCA-NPs + TCA and PTX@GCA-NPs + CHE refer to the rats was orally administered with TCA and subcutaneous injected with CHE before oral PTX@GCA-NPs, respectively. (PTX: 10 mg/kg, TCA: 50 mg/kg, CHE: 3.6 mg/kg). Data is presented as mean ± SEM, n = 3.

| Parameters | Taxol (i.v.) | Taxol (p.o.) | PTX@NPs | PTX@GCA-NPs | PTX@GCA-NPs + TCA | PTX@GCA-NPs + CHE |
| --- | --- | --- | --- | --- | --- | --- |
| AUC_0-∞_ (mg/L*h) | 15941.94 ± 7350.16 | 254.05 ± 16.45 | 1330.96 ± 334.98 | 4942.47 ± 1175.36 | 1819.33 ± 488.22 | 572.77 ± 217.62 |
| C_max_ (mg/L) | 19826.69 ± 12700.31 | 93.19 ± 8.45 | 307.43 ± 39.96 | 1073.1 7± 200.43 | 541.66 ± 381.05 | 96.11 ± 36.76 |
| T_max_ (h) | 0.05 | 1.00 | 1.50 | 1.67 | 2.00 | 1.50 |
| F% |  | 1.59% | 8.35% | **31.00%** | 11.41% | 3.59% |
